# How Do People Experience and Respond to Social Control From Their Partner? Three Daily Diary Studies

**DOI:** 10.3389/fpsyg.2020.613546

**Published:** 2021-01-13

**Authors:** Urte Scholz, Gertraud Stadler, Corina Berli, Janina Lüscher, Nina Knoll

**Affiliations:** ^1^Applied Social and Health Psychology, Department of Psychology, University of Zurich, Zurich, Switzerland; ^2^University Research Priority Program “Dynamics of Healthy Aging,” University of Zurich, Zurich, Switzerland; ^3^Institute Gender in Medicine, Charité – Universitätsmedizin Berlin, Berlin, Germany; ^4^Division of Health Psychology, Department of Education and Psychology, Freie Universität Berlin, Berlin, Germany

**Keywords:** social control, health behavior, behavior change, reactance, affect, daily diary, couples

## Abstract

Positive and negative forms of social control are commonly used to regulate another person’s health-related behaviors, especially in couples. Social control efforts have been shown to result in desirable, but also undesirable effects on different outcomes. Little is known for which outcomes, when, and under which contextual conditions these different effects unfold in people’s everyday lives. Using the dual-effects model of health-related social control, we predicted that same-day and previous-day positive social control would result in desirable effects on target behavior, and same-day positive control on affect. Same-day and previous-day negative control was assumed to result in undesirable effects on reactant responses (i.e., doing the opposite of what the partner wanted and hiding the unhealthy behavior), and same-day negative control on affect. Further, we explored whether it makes a difference if one or both partners intend to change their health behavior. Three daily diary studies addressed these questions for smoking (Studies 1 and 2), and physical activity (Study 3). Receiving more positive control related to more desirable target behavior, and feeling better; more negative control was associated with more reactant responses and feeling worse. Social control unfolded its effects within 1 day, but hardly across days, indicating that control and its reactions to it are fast-acting processes in daily life. The pattern of results were the same for couples with one and both partners intending to change their behavior. Further, results replicated when using partner-reported provided control. Based on these results, social control cannot be unanimously recommended as a behavior change strategy in couples. Future studies should follow up on dyadic and temporal dynamics of social control in couples’ everyday lives in different contexts.

## Introduction

Interest in the role of social relationships for health-related self-regulation has increased in social and health psychology in recent years, with a growing number of publications (e.g., Overall et al., 2009; Fitzsimons et al., 2015; Young et al., 2019). One interpersonal process involved in the social regulation of health behaviors is social control, defined as the influence on and regulation of another person’s behaviors (Lewis and Rook, 1999). Existing evidence on associations of social control with health behaviors and additional outcomes, such as affect or reactant responses, is mostly based on cross-sectional studies, while the few available longitudinal studies mainly speak to differences between persons (Craddock et al., 2015). It is thus largely unknown how and when, and for which outcomes social control effects unfold within individuals over time. Moreover, it is an open question whether effects of social control differ depending on context, i.e., whether one or both partners of a dyad intend to change their health behavior. Three daily diary studies presented in this article set out to address these questions.

### Social Control: Definition, Theoretical Models, Empirical Evidence, and Research Gaps

Changing health behaviors is notoriously difficult, as research on self-regulation has shown (e.g., Sheeran, 2002). Although there is a long tradition of social psychological theories pointing to the importance of close others for self-regulation (van Lange and Rusbult, 2012), this issue has been largely neglected in health-related self-regulation research so far (Fitzsimons et al., 2015). In this report, we will highlight the role of health-related social control for two prominent health behaviors, as well as for affect, and reactant responses.

Health-related social control is defined as a set of specific interpersonal strategies meant to influence and regulate another person’s health behaviors (Lewis et al., 2004; Young et al., 2019). It is distinct from social support (cf. Rook, 1990) that aims at easing a challenging situation in the support recipient (Cohen, 2004). While this distinction is straightforward, partner regulation, a prominent concept from relationship science focusing on romantic relationships (Simpson et al., 2018; Baker and McNulty, 2020) is more closely related to social control. Partner regulation is defined as “an attempt to resolve relationship problems that arise” (p. 5, Baker and McNulty, 2020). Partner regulation and health-related social control thus address different targets. The only overlap between these two constructs occurs when it comes to influencing health behaviors that cause (potential) problems not only for one partner’s health, but also for the relationship. However, even in this area of overlap an important distinction between partner regulation and social control is that a prominent form of cooperative partner regulation is social support (Baker and McNulty, 2020) which again is distinct from social control. Owing to the differences in partner regulation and health-related social control the present work draws primarily on the health-related social control literature.

Several theoretical models on how health-related social control relates to health behavior and additional outcomes have been proposed (Okun et al., 2007), with the modified dual-effects model of social control receiving recent support by a meta-analysis (Craddock et al., 2015). The original dual-effects model postulates beneficial effects of social control on the target behavior, but at the same time costs in the form of reduced positive and increased negative affect in the social control recipient (Okun et al., 2007). The modified version of the dual-effects model distinguishes between positive and negative social control and has been tested in a number of studies (e.g., Lewis and Rook, 1999; Fekete et al., 2006; Scholz et al., 2013).

Positive control has been defined with a negotiation component: Positive control providers try to get recipients to agree with the change, using control strategies such as discussions about the behavior, complimenting recipients on change attempts or using reminders. Negative control in contrast is defined as lacking this negotiation component and as relying on using pressure and inducing guilt instead for making the target person change their health behavior (Lewis et al., 2004).

Empirical evidence indicates that positive control relates to the behaviors targeted at with a moderate-size effect, while negative control is unrelated to behavior (Craddock et al., 2015). Higher negative control was also moderately related to higher negative affect and lower positive affect. Positive control was unrelated to negative affect and moderately related to more positive affect (Craddock et al., 2015).

Recent research on the dual effects model of health-related social control has gone beyond the target behavior and affect as outcomes and additionally included reactant responses. The intertwined model of reactance postulates that reactance comprises negative affect (such as anger) and negative cognition components that are inseparable (Dillard and Shen, 2005; Rains, 2013). Reactance is triggered by a perceived threat to one’s autonomy (Brehm and Brehm, 1981); it is a common response to attempts of external persuasion and regulation (Dillard and Shen, 2005). Responses to social control, such as doing the opposite of what the control provider wanted the recipient to do (e.g., Tucker, 2002) and hiding undesirable behaviors (Tucker and Anders, 2001), can be considered consequences of reactance in that they represent different forms of direct restoration of one’s autonomy (Brehm and Brehm, 1981). An example for doing the opposite of what the control provider wanted is to smoke even more cigarettes, examples for hiding an undesirable behavior is to smoke in hiding or not telling the partner about skipping the exercise class. In health-related social control research, only a minority of studies has considered these additional, reactance-related outcomes of health-related social control (Craddock et al., 2015). Yet, investigating social control effects on these reactance-related responses is central for gaining a more comprehensive understanding of the consequences of social control. This study will address this issue by applying an extended version of the dual-effects model of social control: We relate both positive and negative social control to health behaviors and affect, and also to hiding the behavior and doing the opposite of the control provider’s intention as two reactance-related responses.

### Between-Person and Within-Person Effects of Social Control

Most research on social control has tested hypotheses by focusing on differences between people: If Person A reports a higher level of negative control compared to Person B, Person A will on average also report higher levels of negative affect than Person B. The association between negative social control and negative affect *within* Person A or Person B over time, however, remains unknown (Hamaker, 2012), e.g., that at times of higher social control people will also experience more negative affect. In fact, the within-person association may be zero or even in the opposite direction as the between-person association (Hamaker, 2012). The strong focus on between-person associations is also in contrast to the causal claim most theories, including the dual-effects model of social control, make on associations *within* persons. The majority of studies on health-related social control followed cross-sectional designs and of the longitudinal ones few examined associations at the within-person level (Craddock et al., 2015). Those studies largely confirmed the hypothesized associations of the dual-effects model, but mostly while examining same-day relations (Khan et al., 2013). Consequently, an open question in health-related social control research is how effects of social control unfold over time within individuals. For answering this question, we will focus on micro-time dynamics from 1 day to another to gain a better understanding of the temporal dynamics of social control and its outcomes in people’s everyday lives (e.g., Scholz, 2019).

### Social Control in Context

A further extension of previous research on social control is the inclusion of contextual factors to better understand under which conditions positive and negative social control unfold their beneficial or undesired effects (Tucker, 2002). So far, relationship quality has been studied most frequently as a contextual moderator of social control effects. Couples with higher relationship quality showed more beneficial and less undesirable outcomes of social control than less satisfied couples (Tucker, 2002; Knoll et al., 2012; Scholz et al., 2013). Another context factor that has not yet been addressed in research on effects of social control is the couple constellation with regard to the intended change in the health-related behavior, for instance, whether one or both partners intend to stop smoking or increase their physical activity. Couples with only one partner intending to change their behavior are more likely to rely on one-sided social control receipt and provision than couples with both partners intending to change jointly. This might be related to stronger threats to recipients’ autonomy, and consequently evoke stronger reactance-related responses, more negative affect, and less behavior change. In contrast, couples in which both members want to change their behavior, the joint endeavor might better legitimize the use of positive and negative control strategies, or enable reciprocation among partners (Gleason et al., 2003), thereby potentially buffering detrimental and intensifying beneficial effects of both positive and negative social control on behavior, affect, and reactance-related responses. The present research comprised different couple constellations with regard to individual or joint intended behavior change allowing to inspect different patterns of results.

### Aims of the Present Research

Based on current evidence, it is largely unknown how, when and for which outcomes social control effects unfold within individuals over time. Moreover, it is an open question whether effects of social control differ depending on whether one or both partners of a dyad intend to change their behavior. This article addresses these questions using data from three daily diary studies, focusing on the within-person perspective.

In line with the modified dual-effects model (Butterfield and Lewis, 2002; Lewis et al., 2004; Scholz et al., 2013), we assumed positive associations between positive social control and the target behavior (i.e., less smoking or more physical activity, respectively). We hypothesized an effect of previous day (Hypothesis 1a, H1a) and same day (Hypothesis 1b, H1b) positive control on the target behavior. Positive control strategies are mostly prospective in nature (discussing, persuading, reminding) and may therefore likely unfold their effects from 1 day to the next.

Given the evidence from the dual-effects model, no hypothesis on associations between negative control and target behavior was formed. We refrain from generating explicit hypotheses for these kind of null effects, because proving that there is no or only a trivially small effect would require impossibly large samples (Cohen, 1990) that are beyond the scope of the intensive longitudinal dyadic studies reported here.

In terms of affect, we investigated whether or not people feel better or worse after receiving positive and negative social control from their romantic partners. Affective responses are likely to change quickly with changes in the situation, i.e., when control is present or not (cf. Gross, 2014). Therefore, we primarily assumed short-acting effects of social control on affect within a given day. We expected that people feel better in response to receiving positive control on the same day (Hypothesis 2; H2), and that they feel worse in response to receiving negative control on the same day (Hypothesis 3; H3).

Regarding associations between positive and negative control with reactant responses, we hypothesized that more negative social control received on the previous day (i.e., lagged effect; Hypothesis 4a, H4a) and on the same day (Hypothesis 4b, H4b) would be associated with more reactant responses, i.e., more doing the opposite of what the partner wanted and more hiding the behavior. Receiving high levels of negative social control is assumed to result in immediate and non-volatile reactance (anger and negative cognitions; Dillard and Shen, 2005). Attempting to restore one’s autonomy will take place the same day the reactance is experienced (i.e., same day effect). Further, the higher the level of negative control received the previous day, the higher the likelihood that this restoration of one’s autonomy might continue from the previous day to the next day (i.e., lagged effect). Positive social control strategies, such as discussions with the target person, still leave room for recipients to choose whether or not to adopt the target behavior. Consequently, the recipients of positive social control would experience no or only a weak threat to their autonomy. Therefore, no hypotheses are set for the associations between positive control and reactant responses.

Finally, we will address a gap in the literature, namely how contextual factors might affect associations between social control and its outcomes. Particularly, we will explore whether effects of social control differ depending on whether one or both partners of a dyad intend to change their behavior. This will be examined at an exploratory level as the couple constellations are part of the different studies. Study 1 tests hypotheses in a couple constellation with only one partner intending to change their behavior, whereas Study 2 and 3 examine social control in couples with both partners intending to change their behavior.

Hypotheses tests and exploratory analyses examined received positive and negative social control reported by target persons. Additionally, effects of partner-provided positive and negative control were examined to learn whether results hold across different perspectives and to exclude potential artifacts due to shared method variance.

## Study 1: Smoker-Non-Smoker Couples

Participants were couples with one smoking partner undergoing a quit attempt. Study 1 focuses on 22 end-of-day diary entries (self-set quite date and 21 days after) of 70 smokers who relapsed after their self-set quite date (of a total of 100 quitting smokers). In line with prior research, relapse was defined as having smoked more than five cigarettes since the quit date at the project’s 1 month follow-up (West et al., 2005). The focus was on relapsers only, because during the time of assessment successful quitters smoked no cigarettes after the quit attempt, therefore they did not show within-person variability in smoking and could not contribute to a better understanding of within-person links between social control and smoking, reactance-related outcomes, and affective reactions.

### Methods

#### Procedure and Sample

The data of Study 1 are from the larger project “Dyadic and Individual Regulation to End Chronic Tobacco Use (DIRECT)” funded by the Swiss National Science Foundation (100014_124516/1). Participants were adult smokers intending to quit during the study, and their non-smoking partners (for a full description of this study’s methods please see Ochsner et al., 2014). To be eligible, smokers had to smoke at least one cigarette daily, intend to quit during the study, be married or in a committed heterosexual relationship for at least 12 months, and be living with their non-smoking partner for at least 6 months. Smokers who attended a smoking cessation program, worked shifts of 24 h, were not fluent in German, or were pregnant were not eligible to participate. Couples received (100 Swiss Francs, about $109) for participating in the diary phase. Participants were provided with study smartphones and instructed to complete daily questionnaires within 1 h of going to bed for 22 consecutive days starting at the self-set quit date. Participants were treated in accordance with the ethical guidelines of the Helsinki Declaration (2000).

Participants had a mean age of 39.96 years (*SD* = 10.40), 22 (31.4%) were women. The majority (70%) reported 9 years of schooling and were currently employed (81.4%). Roughly half (55.7%) of the participants had children, with 35 (50%) living with the children in the same household.

#### Measures

Means and standard deviations across the diary phase, as well as the range of the scales, ICCs and number of participants providing data are displayed in Table 1. All item examples are translations from German.

**TABLE 1:** Means, standard deviations, ranges across all diary days, and intraclass correlations (ICC) of main variables in Study 1, Study 2 for women and men separately, and Study 3.

		*M*	*SD*	*SD*	Range	*ICC*	*N*
Study 1	Daily number of cigarettes smoked	7.31	7.71	3.65	0–45	0.80	70
	Affect	0.03	0.62	0.70	−3 to +3	0.24	49
	Doing the opposite	1.75	0.89	0.83	1–6	0.41	49
	Hiding smoking	1.52	0.80	0.83	1–6	0.39	56
	Positive social control	1.36	0.40	0.38	1–4	0.49	70
	Negative social control	1.12	0.21	0.21	1–4	0.45	70
Study 2 women	Daily number of cigarettes smoked	6.08	6.19	2.81	0–40	0.82	59
	Affect	0.17	1.07	0.91	−3 to +3	0.40	19
	Doing the opposite	1.47	0.98	0.79	1–6	0.42	31
	Hiding smoking	1.17	0.41	0.50	1–6	0.36	43
	Positive social control	1.40	0.42	0.41	1–4	0.48	59
	Negative social control	1.09	0.14	0.20	1–4	0.29	59
Study 2 men	Daily number of cigarettes smoked	6.20	5.16	3.16	0–34	0.71	60
	Affect	0.41	0.93	0.85	−3 to +3	0.40	49
	Doing the opposite	1.55	0.83	0.83	1–6	0.47	54
	Hiding smoking	1.35	0.69	0.74	1–6	0.41	59
	Positive social control	1.52	0.45	0.38	1–4	0.48	60
	Negative social control	1.13	0.22	0.22	1–4	0.29	60
Study 3	Daily MVPA (log)	3.52	0.57	0.95	1.6–4.7	0.36	118
	Affect	0.35	0.48	0.87	−3 to +3	0.21	113
	Doing the opposite	1.30	0.44	0.53	1–6	0.39	120
	Hiding inactivity	1.22	0.38	0.37	1–6	0.48	120
	Positive social control	1.42	0.39	0.43	1–4	0.42	120
	Negative social control	1.11	0.20	0.20	1–4	0.47	120

**N = Number of cases. The ICC (intraclass correlation) stands for the amount of between-person variance in relation to total variance (Bolger and Laurenceau, 2013). MVPA = moderate-to-vigorous physical activity; log = log-transformed.**

*Positive and negative social control* were assessed with four items each by Butterfield and Lewis (2002) and adapted to the context of smoking (Ochsner et al., 2015) and daily assessments. A sample item for positive social control is “Today, my partner tried to influence my smoking behavior by trying to persuade me to reduce my smoking or to quit.” A sample item for negative control is “Today my partner tried to influence my smoking behavior by trying to make me feel guilty.” The response format ranged from 1 = never today to 4 = frequently today.

*Partner provided positive and negative control* was assessed with exactly the same items and response format, but from the perspective of the provider (e.g., Today, I tried to influence my partner’s smoking behavior by trying to make him/her feel guilty).

*Target Behavior: Daily number of cigarettes smoked* was assessed by two questions. “Did you smoke today (including only one puff)?” If participants indicated yes, they were asked to report how many cigarettes they had smoked (Heatherton et al., 1991); if they indicated no, daily number of cigarettes smoked was set to 0.

*Affect* after having received control was measured daily with the item: “How did you feel today after your partner tried to influence you this way?” Response options ranged on a seven-point scale from −3 “much worse” via 0 “unchanged” to + 3 “much better.” A further option for participants was to indicate that their partner did not try to influence their smoking behavior today, which was then coded as missing. This resulted in 49 relapsers providing data for this item across the 22 days.

*Reactant responses*. *Doing the opposite of what the partner wanted* was assessed with an item adapted from Tucker and Anders (2001): “Today I did exactly the opposite of what my partner wanted me to do with regard to my smoking.” *Hiding smoking from the partner* was assessed with an item adapted from Tucker and Anders (2001): “Today I hid my smoking from my partner.” For both items, response options ranged from 1 “today not at all” to 6 “today very frequently.” Again, a further option for participants was to indicate that their partner did not try to influence their smoking today, items were then coded as missing. Across 22 days, data for *doing the opposite* were provided by 49 participants; data for *hiding smoking* were provided by 56 participants.

#### Analytic Strategies

We used multilevel modeling to account for the hierarchical data structure, following recommendations by Bolger and Laurenceau (2013). Within-person (Level 1) predictors were person-mean centered. These centered variables provide information on the daily fluctuation around the person-specific mean, testing links between positive and negative social control and the different outcome variables within persons (Level 1). Between-person (Level 2) predictors, i.e., the average scores across the diary days of the respective variables were grand-mean centered at the sample mean. Due to the different number of participants for the outcomes affect, doing the opposite, and hiding in Study 1 and 2, the centering of the between-person variables was adjusted for the subsamples with available data.

To test Hypotheses 1–4, lagged analyses with a lag of 1 day were run. Models included between-person positive and negative control, within-person previous day, and same day positive and negative social control together with previous day’s outcome to predict the same day outcome. Testing effects of previous day positive and negative control on present day’s outcomes allow to establish temporal order. Further, including present day’s positive and negative control as predictors excludes the possibility that effects of previous day positive and negative control on present day’s outcome are artifacts due to strong associations of present day’s positive and negative control with present day’s outcome. Note that predictors are strongly correlated from one day to the next. Similarly, including previous day outcome in the analyses excludes the possibility that effects of previous day positive and negative control on present day outcomes are artificially inflated because of correlations between previous day positive and negative control and previous day outcomes. Due to lagged analyses, the second diary day served as the first outcome day. A linear time trend centered on Day 2 controlled for common time effects. One unit increase in time indicated 1 week representing all diary days (Day 1 = 0, Day 2 = 0.14, …, Day 7 = 1, Day 8 = 1.14, …).

Variables were standardized for better comparability of effects. While it is possible to standardize with the between-person standard deviation as was done here, it is also possible to standardize with the pooled within-person standard deviation or with the individualized standard deviation. For the present studies, standardizing with the between-person standard deviation was chosen for the following reasons: Standardizing with the between-person standard deviation allows comparing effect sizes (a) across the three studies for different couple constellations, (b) within our studies for between- and within-person effects, and (c) with other studies, e.g., from the meta-analyses on social control (Craddock et al., 2015). Although for between- person effects between-SD standardization and for within-person effects the within-SD standardization would have been ideal, using different standard deviations for standardizations would have prevented comparison of the different effect sizes of between- and within-person effects and with other studies. Thus, we standardized all effects with the between-person standard deviation by dividing all variables by their respective between-person standard deviation (*SD*). Of note, the between- and within-person *SD*s were largely comparable in size (see Table 1, and Supplementary Table P1 for provided control). For each *SD* increase in the predictor, the outcome changes in *SD*s as much as the regression weight indicates. This allows interpretation of effect sizes: *b* < 0.3 = small effect, 0.3 ≤ *b* < 0.5 = medium effect, *b* ≥ 0.5 = large effect (Cohen et al., 2003). As daily number of cigarettes smoked is a meaningful metric, this outcome was not standardized in Studies 1 or 2.

*Daily number of cigarettes smoked* (Studies 1 and 2) was a count variable, thus generalized linear mixed Poisson models with a logarithmic link function were used (Xie et al., 2013), resulting in rate ratios as the regression coefficients. Rate ratios (RR) indicate that a one-unit increase in the predictor results in percentage increase (distance above one) or percentage decrease (distance below one) in the criterion (Atkins et al., 2013). For the other three outcomes in Studies 1 and 2 (doing the opposite, hiding, and affect) linear mixed models were run.

For all analyses, a maximal random effects structure was specified (Barr et al., 2013). In case of non-convergence, the random effects structure was successively reduced until convergence was met. For parsimony, we reported results of the Level-1 random effects in the Supplementary Material S1 (and P2 for provided control). Intra-class correlations (ICC) for all measures were computed. The ICC is the amount of variance between second-level units, in our case persons, in relation to total variance (Bolger and Laurenceau, 2013). All analyses were conducted in SPSS 23, with a probability level of *p* = 0.05.

Sensitivity analyses were conducted for all models. We adjusted for nicotine dependence at baseline (Fagerström test of nicotine dependence; Heatherton et al., 1991), age, and gender in Study 1 and nicotine dependence and age in Study 2 (where we ran separate analyses for men and women). In Study 3, we adjusted for gender, age, BMI, intervention group vs. control group, intervention phase vs. follow-up phase, and weekday vs. weekend, and weartime of accelerometers in the analyses of physical activity. For parsimony, we report the unadjusted analyses in this article. Tables depicting sensitivity analyses are reported in the Supplementary Material S2 (and Supplementary Table P2 for provided control).

#### Results Study 1

Results of Study 1 are displayed in Table 2. For random effects see Supplementary Tables S1-1. Results of partner-reported provided social control, largely reflecting the results of received social control, are displayed in Supplementary Table P1-1. Sensitivity analyses showed the same patterns of results, see Supplementary Table S2-1 for received and Supplementary Table P2-1 for provided control.

**TABLE 2:** Study 1: within- and between-person effects of negative and positive social control on daily number of cigarettes smoked, affect, doing the opposite, and hiding smoking after the quit date for relapsing smokers.

	DV: Number of cigarettes smoked				DV: Affect			DV: Doing the opposite			DV: Hiding smoking		
				
			95% *CI*			95% *CI*			95% *CI*			95% *CI*	
									
Fixed effects	*b*	*RR*	*LL*	*UL*	β	*LL*	*UL*	β	*LL*	*UL*	β	*LL*	*UL*
Intercept	1.25	3.48**	2.52	4.80	0.20	–0.25	0.66	1.29**	1.09	1.49	1.85**	1.38	2.32
Time	0.10	1.10**	1.03	1.19	–0.06	–0.35	0.23	0.43**	0.14	0.72	0.06	–0.25	0.36
Previous day outcome	0.002	1.00	0.90	1.12	−0.34**	–0.49	–0.20	−0.30**	–0.40	–0.20	−0.26**	–0.42	–0.09
**Negative control**													
*Within-person effects*													
On the same day	0.02	1.02	0.97	1.08	−0.30**	–0.41	–0.19	0.18*	0.04	0.32	0.11*	0.02	0.21
On the previous day	0.002	1.00	0.95	1.05	–0.10	–0.21	0.02	0.004	–0.11	0.12	0.07	–0.02	0.17
Between-person effects	0.36	1.43*	1.07	1.89	–0.18	–0.56	0.21	0.22	–0.05	0.49	0.30	–0.12	0.72
**Positive control**													
*Within-person effects*													
On the same day	–0.05	0.95*	0.91	0.99	0.35**	0.22	0.48	–0.07	–0.16	0.02	−0.11*	–0.20	–0.01
On the previous day	–0.05	0.96	0.91	1.0	0.22**	0.08	0.36	–0.01	–0.11	0.09	–0.05	–0.18	0.08
Between-person effects	–0.19	0.83	0.60	1.14	0.40*	0.06	0.74	–0.09	–0.32	0.14	0.19	–0.18	0.57

For the effects of all random effects please see Supplementary Table S1-1. For daily number of cigarettes smoked: n = 70, n = 1,186 available days; for affect: n = 37; n = 353 available days; for doing the opposite: n = 38, n = 357 available days; for hiding: n = 48, n = 530 available days; RR = rate ratio; b = unstandardized regression coefficients (outcome in original metric), β = standardized regression coefficients (predictor and outcome in between-person SD units), 95% CI = 95% confidence interval; LL = lower level; UL = upper level; * p < 0.05, ** p < 0.01.

#### Does Positive Social Control Predict Daily Number of Cigarettes Smoked (H1a and H1b)?

In contrast to H1a assuming a previous-day effect, but in support of H1b assuming a same-day effect, positive control on the same day, but not the previous day was significantly related to smoking: On days with higher than usual same-day positive control smokers reported 5% less cigarettes smoked (see Table 2). As expected from the theoretical assumptions of the extended dual-effects model, same day and previous day negative control were unrelated to daily number of cigarettes smoked.

#### Does Positive and Negative Social Control Predict Affect (H2 and H3)?

In support of H2, same day positive control was associated with feeling better. Additionally, a positive effect of previous-day positive control on feeling better emerged. In line with H3, same-day, but not previous-day negative control was related to smokers feeling worse.

#### Does Negative Social Control Predict More Reactant Responses (H4a and H4b)?

Supporting H4b, but contrasting H4a, only same day, but not previous day negative control was related to *doing the opposite*, indicating that higher than usual levels of negative control were related to more doing the opposite within the same day. Similarly, only same day but not previous day negative control significantly predicted *hiding*: More than usual same day negative control related to more hiding. Further, same day positive control was significantly related to less hiding, but –as expected from the extended dual-effects model- unrelated to doing the opposite.

#### Brief Discussion of Study 1 Results

Overall, results mainly confirmed our hypotheses on same-day effects (see Table 3 and Supplementary Table P6 for provided control for a color-coded overview of the results across all studies with regard to confirmation/disconfirmation of Hypotheses 1–4). Positive control predicted fewer cigarettes smoked (H1b) and less hiding. Negative control predicted more doing the opposite and hiding (H4b). However, these effects emerged only for the same day, but not the previous day. This speaks in favor of social control being a fast process linked with rather immediate outcomes in people’s everyday lives. For the reactant responses this might be explained by relationship maintenance issues: displaying maladaptive behaviors across several days may prove negative for the relationship (Burke and Segrin, 2017). Thus, smokers might try to avoid prolonged negative behavioral reactions for the sake of their relational well-being.

**TABLE 3:** Overview of results for study 1, study 2 (for men and women), and study 3 for received positive and negative social control.

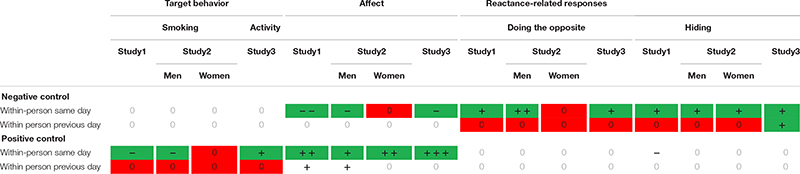

**0 = non-significant effect; + = positive significant effect; − = negative significant effect; + or − = small effect size, + + or − − = medium effect size, +++ = large effect size; effect sizes are only displayed for significant effects; red color coding: disconfirming hypothesis, green color coding: confirming hypothesis; white color coding: no a priori hypotheses.**

Hypotheses 2 and 3 for affect were largely confirmed and highlight the applicability of the assumptions on affective correlates in the extended dual-effects model to smokers’ everyday lives. The more consistent link of affective reactions to receiving positive and negative control compared to the behavioral outcomes might also be explained by better measurement precision: Affect was assessed as a direct affective reaction to receiving control from the partner. All behavioral outcomes were assessed in a less targeted manner, leaving room for other influences.

The results of Study 1 emerged in a couple constellation with only one partner intending to change their behavior. Studies 2 and 3 addressed the same hypotheses in the context of both partners intending to change their behavior.

## Study 2: Dual-Smoker Couples

The second study had the same research design as Study 1, but participants were dual-smoker couples intending to quit jointly on a self-set quit date. This allows for a comparison between effects of social control in a different couple context. Due to this planned comparison, data for female and male smokers were analyzed separately to ensure the same structure of results as in Study 1.

### Methods

#### Procedure and Sample

Data from this study came from the larger project “Individual regulation and dyadic exchanges during an on-going quit attempt in dual-smoker couples” funded by the Swiss National Science Foundation (PP00P1_133632/1). The procedure and eligibility criteria were the same as in Study 1 with the exception that Study 2 focused on dual-smoker couples with both partners intending to quit jointly during the study (Lüscher et al., 2017; for a comprehensive description of the study’s procedures, etc., please see Lüscher and Scholz, 2017). The project was approved by the Ethics Committee of the Faculty of Human Sciences of the University of Bern in Switzerland (2011-11-14409).

Like Study 1, the current study focused on 22 end-of-day diary entries (self-set quite date and 21 days after) of *N* = 60 male smokers and *N* = 59 female smokers who relapsed after a joint self-set quite date (of a total of 83 couples participating in the diary phase). In line with Study 1, relapse was defined as having smoked more than five cigarettes since quit date at the project’s 1 month follow-up (West et al., 2005).

Female smokers had a mean age of 38.53 (*SD* = 14.95). Most (*n* = 26, 44.1%) reported 9 years of schooling and a majority of 64.4% (*n* = 38) were currently employed. Male smokers had a mean age of 40.85 (*SD* = 14.65). The majority (*n* = 33, 55%) reported 9 years of schooling and were currently employed (*n* = 46, 76.7%). Of all couples, 26 (43.3%) were married, and 11 (18.3%) couples had at least one child living in their household.

#### Measures

All constructs were assessed with the same items as in Study 1. All descriptive information on the measures are provided in Table 1 (and Supplementary Table P1 for provided control).

#### Results of Study 2

Results of Study 2 are displayed in Table 4 (male smokers) and Table 5 (female smokers). For random effects see Supplementary Tables S1-2, S1-3. Results of partner-reported provided control, again largely reflecting results of received social control, are reported in Supplementary Tables P1-2, P1-3. For sensitivity analyses also resulting in the same patterns of results, please see Supplementary Tables S2-2, S2-3 for received and Supplementary Tables P2-2, P2-3 for provided control.

**TABLE 4:** Study 2 men: within- and between-person effects of negative and positive social control on daily number of cigarettes smoked, affect, doing the opposite, and hiding smoking after the quit date for relapsing smokers.

	DV: Number of cigarettes smoked				DV: Affect			DV: Doing the opposite			DV: Hiding smoking		
				
			95% *CI*			95% *CI*			95% *CI*			95% *CI*	
									
Fixed effects	*b*	*RR*	*LL*	*UL*	β	*LL*	*UL*	β	*LL*	*UL*	β	*LL*	*UL*
Intercept	1.23	3.43**	2.42	4.86	0.38*	0.03	0.73	1.82**	1.51	2.13	2.03**	1.70	2.36
Time	0.07	1.07	0.94	1.22	–0.14	–0.38	0.10	0.06	–0.06	0.17	–0.03	–0.22	0.16
Previous day outcome	–0.02	0.98	0.91	1.06	−0.21**	–0.30	–0.13	−0.20**	–0.33	–0.08	−0.28**	–0.37	–0.18
**Negative Control**													
*Within-person effects*													
On the same day	0.01	1.01	0.98	1.05	−0.18*	–0.32	–0.03	0.30**	0.22	0.38	0.24**	0.08	0.39
On the previous day	–0.004	1.00	0.97	1.02	–0.04	–0.11	0.03	0.04	–0.05	0.12	0.05	–0.03	0.13
Between-person effects	–0.11	0.90	0.72	1.11	–0.05	–0.29	0.18	0.17	–0.11	0.46	0.74**	0.54	0.94
**Positive control**													
*Within-person effects*													
On the same day	–0.05	0.96*	0.92	0.99	0.22*	0.05	0.39	–0.08	–0.23	0.07	–0.10	–0.27	0.07
On the previous day	–0.02	0.98	0.96	1.0	0.09*	0.01	0.17	–0.06	–0.15	0.04	–0.07	–0.16	0.02
Between-person effects	0.22	1.24	0.93	1.66	0.30*	0.04	0.56	0.55**	0.25	0.84	–0.16	–0.37	0.04

**For the effects of all random effects please see Supplementary Table S1-2. For daily number of cigarettes smoked: n = 60, n = 945 available days; for affect: n = 38; n = 480 available days; for doing the opposite n = 49, n = 620 available days; for hiding: n = 56, n = 777 available days; RR = rate ratio b = unstandardized regression coefficients (outcome in original metric), β = standardized regression coefficients (predictor and outcome in between-person SD units), 95% CI = 95% confidence interval; LL = lower level; UL = upper level; ^∗^p < 0.05, ^∗∗^p < 0.01.**

**TABLE 5:** Study 2 women: within- and between-person effects of negative and positive social control on daily number of cigarettes smoked, affect, doing the opposite, and hiding smoking after the quit date for relapsing smokers.

	DV: Number of cigarettes smoked				DV: Affect			DV: Doing the opposite			DV: Hiding smoking		
				
			95% *CI*			95% *CI*			95% *CI*			95% *CI*	
									
Fixed effects	*b*	*RR*	*LL*	*UL*	β	*LL*	*UL*	β	*LL*	*UL*	β	*LL*	*UL*
Intercept	1.19	3.28**	2.27	4.74	0.26*	0.03	0.49	1.22**	0.98	1.45	2.89**	2.62	3.15
Time	–0.05	0.96	0.84	1.10	–0.05	–0.15	0.05	0.09	–0.02	0.19	0.02	–0.17	0.22
Previous day outcome	–0.03	0.97	0.91	1.03	–0.02	–0.11	0.08	−0.07*	–0.13	–0.01	−0.15*	–0.27	–0.03
**Negative control**													
*Within-person effects*													
On the same day	0.01	1.0	0.98	1.04	–0.09	–0.20	0.03	0.09	–0.04	0.22	0.16*	0.001	0.32
On the previous day	0.01	1.0	0.98	1.03	0.002	–0.05	0.05	0.02	–0.02	0.07	0.06	–0.003	0.13
Between-person effects	–0.17	0.85	0.66	1.09	–0.15	–0.32	0.02	0.02	–0.17	0.22	0.48**	0.25	0.71
**Positive control**													
*Within-person effects*													
On the same day	–0.03	0.98	0.94	1.01	0.37**	0.21	0.53	0.01	–0.06	0.07	–0.01	–0.09	0.08
On the previous day	–0.03	0.97	0.92	1.03	0.01	–0.07	0.10	–0.02	–0.09	0.04	0.04	–0.04	0.12
Between-person effects	0.23	1.26	0.99	1.61	0.28**	0.12	0.45	0.12	–0.08	0.31	–0.14	–0.35	0.07

**For the effects of all random effects please see Supplementary Table S1-3. For daily number of cigarettes smoked: n = 59, n = 986 available days; for affect: n = 32; n = 336 available days; for doing the opposite: n = 50, n = 606 available days; for hiding: n = 55, n = 820 available days; RR = rate ratio b = unstandardized regression coefficients (outcome in original metric), β = standardized regression coefficients (predictor and outcome in between-person SD units), 95% CI = 95% confidence interval; LL = lower level; UL = upper level; ^∗^p < 0.05, ^∗∗^p < 0.01.**

#### Does Positive Social Control Predict Daily Number of Cigarettes Smoked (H1)?

Comparable to results of Study 1, and only supporting H1b, it was only same, but not previous day positive control that was significantly related to smoking and only in men: On days with higher than usual same day positive control male smokers reported 4% less cigarettes smoked. In line with the extended dual-effects model, no significant effects emerged for previous or same day negative social control for male and female relapsers.

#### Does Positive and Negative Social Control Predict Affect (H2 and H3)?

H2 was fully supported in both the female and the male subsample: for both women and men, same day positive control was related to feeling better. Additionally, for men only previous day positive control was associated with feeling better. Supporting H3 in the male sample, same-, but not previous day negative control received from the partner was related to feeling worse. For women neither same nor previous day negative control was associated with affect, partly disconfirming H3.

#### Does Negative Social Control Predict Daily Reactant Responses (H4)?

Partly in line with H4b, but disconfirming H4a, the only significant effect emerged for same day negative control in male smokers, indicating that at higher than usual levels of same day negative control, higher levels of doing the opposite were reported by men. Again, in line with H4b, but disconfirming H4a, same-, but not previous day negative control significantly predicted hiding: In men and women alike, more than usual same day negative control was associated with more hiding. In accordance with the extended dual-effects model, no effects of same- or previous day positive control on hiding emerged.

#### Brief Discussion of Study 2 Results

As in Study 1, analyses largely supported our hypotheses on same-day effects (see Table 3 and Supplementary Table P6 for provided control for the color-coded overview). Overall, the pattern of results in dual-smoker couples with the joint goal of quitting smoking was very similar to the pattern of smokers within smoker–non-smoker couples, i.e., with only one partner intending to change their behavior. Study 2 replicates the majority of results of Study 1 with regard to effect sizes. This might be explained by a strong influence of the target behavior: quitting smoking might be such a desired behavior change that it legitimizes many means to be achieved. One limitation of Study 1 and 2 were the use of self-reported target behavior. Study 3 addresses this shortcoming by assessing behavior (physical activity) objectively. Moreover, Study 3 introduces another context by focusing on couples where both partners were overweight and intended to increase their daily physical activity. Thus, instead of giving up an undesirable behavior as in studies 1 and 2, Study 3 focuses on the uptake of a desirable behavior, i.e., more physical activity.

## Study 3: Overweight Couples Increasing Their Physical Activity

Overweight (i.e., with a Body Mass Index, BMI, higher than 25) individuals are particularly encouraged to engage in regular physical activity for weight regulation and health benefits (World Health Organization, 2004). The World Health Organization recommends adults to engage in at least 150 min of moderate- or 75 min of vigorous-intensity aerobic activity or a combination thereof per week. A recent meta-analysis indicates promising effects of couple-oriented interventions on physical activity (Richards et al., 2017). In Study 3, participating couples were characterized by both partners being overweight and insufficiently physically active, but both intending to increase their physical activity. Thus, similar to Study 2, both partners intended to change their behavior. Comparing results across the three different studies exploratorily will thus provide a comprehensive picture for different couple constellations across different behaviors.

### Methods

#### Procedure and Sample

The data of Study 3 came from the larger project “A Dyadic Action Control Trial in Overweight and Obese Couples (DYACTIC)” funded by the Swiss National Science Foundation (PP00P1_133632/1) and registered as a randomized controlled trial (ISRCTN15705531). The study was approved by the Ethics Committee of the Faculty of Human Sciences of the University of Bern, Switzerland (2011-12-36206). Analyses of the current study are secondary analyses unrelated to the intervention. The intervention conditions were included in all analyses as a covariate, but were not the focus of the research question (for a full description of this study’s design, recruitment procedures, and primary analyses on the effectiveness of the intervention see Berli et al., 2016). Part of the intervention was to randomly assign one of the partners to be the target person for behavior change. Only target persons reported on the social control received from their partners, partners reported on the social control provided to target persons.

Criteria for participation were that both partners of eligible heterosexual couples had to be between 18 and 75 years old, both partners had to be overweight (body mass index, BMI > 25), insufficiently physically active (< 30 min per day of moderate-to-vigorous physical activity, MVPA), and both had to intend to enhance their physical activity. As in studies 1 and 2, eligible couples had to live in a committed relationship for at least 12 months and cohabit for at least 6 months and to be fluent in German. Moreover, for reasons related to the intervention, couples were only eligible if able to receive and read text messages throughout the day. Exclusion criteria were: working 24 h shifts, participating in a professional weight loss program during the time of the study, and pregnancy in women.

Couples were provided with study smartphones and accelerometers. The end-of-day diary phase started after a baseline assessment and comprised 28 consecutive days. Analyses focus on the randomly assigned target persons of the intervention only. Couples received a financial incentive of (100 Swiss Francs; about $109) for completing the diary phase.

A total of *N* = 120 target persons and their partners participated in the diary phase of this study^1^. Participants had a mean age of 46.03 (*SD* = 13.64), *n* = 62 (51.7%) were women. The majority of participants (*n* = 70, 58.3%) reported 9 years of schooling and were currently employed (*n* = 78, 65%). Of *n* = 69 (57.5%) reporting to have children, *n* = 52 (43.3%) also lived with children in the same household. BMI of participants was *M* = 31.01 (*SD* = 5.6; range = 24.98–61.73).

#### Measures

Means and standard deviations across the diary phase, as well as the theoretical range of the scales, ICCs and number of participants providing data are displayed in Table 1 (and Table P1 in the Supplementary Material for provided control). All item examples are translations from German.

*Positive and negative social control* were assessed with four items each from Butterfield and Lewis (2002) and adapted to the context of physical activity in daily assessments. A sample item for positive social control reads “Today, my partner tried to positively influence my physical activity by stating how important it is to him/her that I am physically active.” A sample item for negative control is “Today my partner tried to positively influence my physical activity by trying to make me feel guilty.” The response format ranged from 1 = never today to 4 = frequently today.

*Partner provided positive and negative control* was assessed by the same items and response format asked from the provider perspective (e.g., Today, I tried to positively influence my partner’s physical activity by stating how important it is to me that he/she is physically active.).

*Target behavior: Moderate-to-vigorous physical activity (MVPA)* was assessed across the 28 days with a triaxial accelerometer monitoring device (GT3X +, ActiGraph, Pensacola, FL) worn at the hip on the side of the dominant hand during waking hours. The GT3X + measures physical activity reliably and validly (Sasaki et al., 2011). Non-wear time was assessed and filtered in the analyses using an automated algorithm based on 90 min of consecutive zeros in vector magnitude counts per minute (cpm) (Choi et al., 2011). Only days with a minimum of 10 h of wear time (Colley et al., 2010) were included in the analyses. Further, weartime of the accelerometer data was controlled for in all analyses on MVPA. For each participant, total minutes in MVPA per day were calculated based on the threshold of 2,690 cpm in vector magnitude (Sasaki et al., 2011), resulting in overall daily MVPA in minutes. The final variable was log transformed, as the distribution of the variable was strongly skewed. A total of *N* = 119 participants provided accelerometer-based physical activity data.

*Affect* after having received control was measured daily with the item: “How did you feel today after your partner tried to influence you this way?” Seven response options ranged from −3 “much worse” via 0 “unchanged” to + 3 “much better.” Also, participants could indicate that their partner had not tried to influence their physical activity that day (coded “missing”), resulting in 113 participants providing data for this outcome across 28 days.

*Reactant responses. Doing the opposite of what the partner wanted* was assessed daily by an item adapted from Tucker and Anders (2001): “Today I did exactly the opposite of what my partner wanted me to do with regard to my physical activity.”

*Hiding was* assessed by an item adapted from Tucker and Anders (2001): “Today I hid from my partner that I was not physically active.” Response options for both items ranged from 1 “today not at all true” to 6 “today very true.” All (*N* = 120) participants reported on these items across 28 days.

#### Results of Study 3

Results of Study 3 are displayed in Table 6. For random effects see also Supplementary Tables S1-4. In Study 3, effects of provided control paralleled effects of received control for MVPA and affect, but not for the two reactant responses (see Supplementary Table P1-4). Sensitivity analyses showed the same patterns of results, see Supplementary Table S2-4 for received and Supplementary Table P2-4 for provided control.

**TABLE 6:** Study 3: within- and between-person effects of negative and positive social control on physical activity (MVPA), affect, doing the opposite, and hiding inactivity.

	DV: MVPA			DV: Affect			DV: Doing the opposite			DV: Hiding inactivity		
				
		95% *CI*			95% *CI*			95% *CI*			95% *CI*	
								
Fixed effects	β	*LL*	*UL*	β	*LL*	*UL*	β	*LL*	*UL*	β	*LL*	*UL*
Intercept	6.10**	5.82	6.37	0.50**	0.25	0.74	2.93**	2.69	3.17	3.23**	3.02	3.44
Time	–0.03	–0.14	0.07	0.005	–0.12	0.13	–0.01	–0.10	0.07	0.04	–0.03	0.11
Previous day outcome	−0.17**	–0.22	–0.12	−0.08*	–0.14	–0.01	–0.04	–0.10	0.01	−0.08*	–0.14	–0.01
**Negative control**												
*Within-person effects*												
On the same day	–0.04	–0.10	0.03	−0.23**	–0.36	–0.10	0.14**	0.05	0.23	0.11**	0.07	0.15
On the previous day	–0.01	–0.07	0.05	–0.04	–0.10	0.03	–0.01	–0.06	0.05	0.06**	0.03	0.10
Between-person effects	–0.01	–0.24	0.22	−0.18*	–0.33	–0.03	0.60**	0.43	0.77	0.49**	0.34	0.65
**Positive control**												
*Within-person effects*												
On the same day	0.14**	0.07	0.21	0.57**	0.44	0.69	–0.001	–0.08	0.07	0.005	–0.05	0.06
On the previous day	0.02	–0.03	0.07	–0.001	–0.07	0.07	–0.004	–0.05	0.04	0.004	–0.03	0.04
Between-person effects	–0.09	–0.30	0.12	0.47**	0.31	0.63	0.07	–0.11	0.25	0.02	–0.15	0.18

**For the effects of the control variables as well as all random effects please see Supplementary Table S1-4. For MVPA: n = 117, n = 2,326 available days; for affect: n = 101; n = 1,697 available days; for doing the opposite n = 120, n = 2,918 available days; for hiding: n = 120, n = 2,918 available days; β = standardized regression coefficients (predictor and outcome in between-person SD units), 95% CI = 95% confidence interval; LL = lower level; UL = upper level; ^∗^p < 0.05, ^∗∗^p < 0.01.**

#### Does Positive Social Control Predict Daily Physical Activity (H1)?

Supporting H1b, but disconfirming H1a, same day positive social control, but not previous day positive social control significantly predicted daily MVPA. That is, one standard deviation higher than usual same day positive control was related to 0.14 standard deviations more MVPA, indicating a small effect. No significant effects emerged for previous or same day negative social control on MVPA as would have been expected from the extended dual-effects model.

#### Does Positive and Negative Social Control Predict Affect (H2 and H3)?

Confirming H2 and H3, same-, but not previous day negative or positive social control were related to affect: More than usual same day negative control was associated with feeling worse, more than usual same day positive control was associated with feeling better.

#### Does Negative Social Control Predict Daily Reactance-Related Responses (H4)?

Supporting H4b, but disconfirming H4a, same-, but not previous day negative control was related to more doing the opposite. For hiding, both H4a and H4b could be confirmed: more than usual previous- and same day negative social control were associated with more hiding. As expected from the theoretical assumptions of the extended dual-effects model, previous-day and same-day positive social control were unrelated to the reactant responses.

#### Brief Discussion of Study 3 Results

In Study 3 we examined hypotheses in the context of a desirable target behavior, i.e., increasing regular physical activity. Previous research on social control did not find substantial differences with regard to frequency or general impact of social control on desirable and undesirable behaviors (Lewis and Rook, 1999). Results of Study 3 further added to this evidence base by using an objective measure of behavior. The positive association with same day positive control partly confirming H1 is thus net of shared method variance.

The overall pattern of results of Study 3 replicated findings of studies 1 and 2 (see Table 3 and Supplementary Table P6 for provided control for an overview of the pattern of results). This is notable because Study 3, just like Study 2, but different than Study 1, examined associations in the context of both partners intending to change their behaviors but with another behavior.

## General Discussion

In the present article, we investigated how and when negative and positive social control would unfold desirable and less desirable effects for which outcomes in romantic partners. We also compared patterns of social control findings between couples with one or both partners wanting to change their behavior.

Across three studies, our research confirms many of our hypotheses based on the modified dual-effects model of social control (Butterfield and Lewis, 2002; Scholz et al., 2013; Craddock et al., 2015) at the daily level: Receiving higher than usual levels of positive social control on a specific day was related to less smoking and more physical activity on the same day (H1b) and to feeling better on the same day (H2). Receiving higher than usual levels of negative control on a specific day was associated with feeling worse on the same day (H3) and with more reactant responses on the same day (doing the opposite and hiding; H4b). It is noteworthy that the within-person effects were in part medium to large in size.

In contrast to our assumptions, only one out of twelve hypothesized effects of previous day positive control on target behavior and of previous day negative control on reactant responses emerged. Hypotheses on the assumed lagged effects (H1a and H4a) were thus disconfirmed. Consequently, results of the present studies indicate that social control is a fast process unfolding its effects within 1 day, but hardly across days. The rationale for assuming lagged effects on future behavior was the prospective nature of positive control and prolonged need for restoration of one’s autonomy after receiving high levels of negative control. It seems, however, that this all happened within a day. The lack of lagged effects might be due to the relatively low levels of social control receipt which is commonly reported in studies on social control (e.g., Hohl et al., 2018; Khan et al., 2013). These rather low levels of control might not have triggered strong reactant responses in recipients or made the targeted behavior change last longer than 1 day. Given the results on the regulation of the provision of social control in the service of relational well-being (Burke and Segrin, 2017), it is also possible that days high in social control were rather followed by days low in social control. Thereby also easing the need to react in a reactant way. Moreover, control recipients might apply relationship maintenance strategies that keep them from reacting too negatively to the receipt of negative control from their partner (Stafford, 2011).

Generally, developing theories on timing of effects that provide a rationale for when and for how long effects occur is necessary (cf. Scholz, 2019). Future studies are thus needed that apply a mixed methods approach that addresses questions on the provision/receipt and duration of effects of social control in couples with one or both partners changing their behavior. Audio recordings of real life conversations combined with the coding of positive and negative control (Badr et al., 2015) would constitute a fine-grained and observational approach to inform theory building of timing of control and its effects.

Effects of received control were largely confirmed by analyses with provided social control. This is noteworthy as it excludes the possible explanation that the results involving received control might mainly be due to shared method variance for all outcomes but the objectively measured MVPA in Study 3; thus substantially strengthening the validity of the results. The only apparent discrepancy between the results of *received* and partner-reported *provided* control, regarding the reactant outcomes, was present in Study 3. Possibly, and in line with theoretical assumptions on reactance, the subjective feelings of threatened autonomy through the receipt of social control is crucial and might thus not have been present for provided control.

All three studies used an intensive longitudinal design allowing to examine processes within persons. The within-person results are of special importance: They are based on individual daily experiences and thus indicate the relevance of social control receipt for how each individual behaves and feels. Given that the majority of studies on health-related social control are still following a cross-sectional design limiting the conclusions drawn to differences between persons only (cf. Craddock et al., 2015), this study furthers our knowledge by showing meaningful effects over time within persons.

Finally, patterns of results across all three studies were strikingly similar, indicating that affective and behavioral correlates might be basically the same for control recipients across different couple constellations (i.e., only one or both partners intending to change their behavior) and different health behaviors. One reason for this consistent pattern could be that couples shared the goal for behavior change in all three studies: Partners were rooting for the target persons to succeed in their smoking quit attempt and in increasing their physical activity. Sharing a goal *per se*, independent from the target of this goal, be it one partner or the dyad, may legitimize the means both partners apply to reach this goal. Transactive goal dynamics theory, a theory defining the dyad as unit of analysis (Fitzsimons et al., 2015), provides a framework for different dyadic goal constellations and their assumed consequences for goal pursuit. Future studies should use this theory for assessing goal orientation in couples directly in order to allow examining its consequences more explicitly. In addition, other contextual factors, such as gender, age, or type of dyad (e.g., romantic couples, friends, family) and their potential moderating role of social control effects deserve more systematic investigation.

### Limitations and Outlook

The results of these three studies need to be interpreted while keeping several limitations in mind. The three intensive longitudinal studies allow conclusions about temporal processes, with the within-person effects of social control indicating relatively fast processes. We have included time in all models thus ruling out time as a third variable explaining the within-person effects of social control on outcomes. However, these studies cannot establish causality, as time-varying covariates such as daily stressors could still provide a third-variable explanation for these within-person effects. It could also be the case that the social control-behavior relationship is reciprocal or that control recipients’ behavior trigger the provision of social control.

Moreover, because lagged effects can be assumed to be smaller than same-day effects to begin with, power issues might have played a role. As these are among the first studies to examine lagged effects of social control on different outcomes at the within-person level, no *a priori* power analysis was possible (cf. Bolger and Laurenceau, 2013). Future lab experiments and real-life intensive longitudinal field experiments could increase positive social control and decrease negative social control to help establish causality. Furthermore, it is not clear how generalizable the current findings are for people intending to change other health-relevant behaviors and if there are moderators of these effects. The theories of social control claim universal applicability across behaviors (Okun et al., 2007). Consequently, there is also no empirical examination of differences between social control effects for different behaviors. Yet, it is possible that there are certain behaviors that are particularly sensitive to control and thus responses to social control attempts could be more pronounced in these behavioral domains. One example is a study on college students’ reports of weight-related social control showing adverse effects on several outcomes particularly for young women but not for men (Brunson et al., 2014). This further emphasizes the need for better understanding not only how and when, and for which outcomes, but also for which behaviors and for whom social control unfolds its effects over time. Whereas the present study was able to contribute to some of these open questions, more systematic and particularly comparative research in different behavioral domains, contexts, and time frames on social control effects is still needed.

## Conclusion

Social control aims at promoting another person’s behavior change. The results of our studies demonstrated that positive control on a given day was related to target behaviors on that same day, but that negative social control was not. And whereas receiving more positive social control related to feeling better, more negative social control was associated with feeling worse and with more reactant responses. Thus, based on the present findings, only positive social control can be recommended as a strategy for inducing behavior change in another person. Moreover, our studies demonstrated that social control unfolds its effects within 1 day, but not across days, indicating that control and its outcomes are fast-acting processes. Different dyadic constellations where one or both partners intended to change their behavior did not make a difference for processes over time. Future studies should follow up on dyadic and temporal dynamics of negative and positive social control in couples’ everyday lives, paying special attention to theory development on timing of effects and time-varying changes in contexts of social control.

## Data Availability Statement

The raw data supporting the conclusions of this article will be made available by the authors, without undue reservation, to any qualified researcher.

## Ethics Statement

The studies involving human participants were reviewed and approved by Study 1: Ethics Checklist of the Ethics Committee of the Faculty of Arts and Social Sciences of the University of Zurich, Switzerland; Studies 2 and 3: Ethics Committee of the University of Bern’s Faculty of Human Sciences in Switzerland (2011-11-14409 and 2011-12-36206). The patients/participants provided their written informed consent to participate in this study.

## Author Contributions

US analyzed the data and wrote the manuscript. GS supported data analyses. GS, CB, JL, and NK provided extensive feedback to the manuscript. JL collected data of Study 2. CB collected data of Study 3. All authors approved the final version of the manuscript for submission and publication.

## Conflict of Interest

The authors declare that the research was conducted in the absence of any commercial or financial relationships that could be construed as a potential conflict of interest.

## Footnotes

1 *N* = 123 couples were randomized to the different intervention conditions, *N* = 2 did not show up for baseline, *N* = 1 provided no diary data. Thus, the final sample of this study was 120 participants.
