# Supplementary material for: How Do People Experience and Respond to Social Control From Their Partner? Three Daily Diary Studies
Source: Front Psychol. 2021 Jan 13;11:613546. doi: 10.3389/fpsyg.2020.613546 (PMC7838347; doi:10.3389/fpsyg.2020.613546)
Supplement: Supplementary file 1 [file Table_1.pdf]

Supplemental Material S1

Table S1-1;

Study 1: Within- and Between-Person Effects of Negative and Positive Social Control on Daily Number of Cigarettes Smoked, Affect, Doing the Opposite, and Hiding Smoking After the Quit Date for Relapsing Smokers Including Random Effects

	DV: Number of cigarettes smoked				DV: Affect			DV: Doing the opposite			DV: Hiding smoking		
			95% *CI*			95% *CI*			95% *CI*			95% *CI*	
Fixed Effects	*b*	*RR*	*LL*	*UL*	β	*LL*	*UL*	β	*LL*	*UL*	β	*LL*	*UL*
Intercept	1.25	3.48**	2.52	4.80	0.20	-0.25	0.66	1.29**	1.09	1.49	1.85**	1.38	2.32
Time	0.10	1.10**	1.03	1.19	-0.06	-0.35	0.23	0.43**	0.14	0.72	0.06	-0.25	0.36
Previous day outcome	0.002	1.00	0.90	1.12	-0.34**	-0.49	-0.20	-0.30**	-0.40	-0.20	-0.26**	-0.42	-0.09
Negative control													
Within-person effects													
On the same day	0.02	1.02	0.97	1.08	-0.30**	-0.41	-0.19	0.18*	0.04	0.32	0.11*	0.02	0.21
On the previous day	0.002	1.00	0.95	1.05	-0.10	-0.21	0.02	0.004	-0.11	0.12	0.07	-0.02	0.17
Between-person effects	0.36	1.43*	1.07	1.89	-0.18	-0.56	0.21	0.22	-0.05	0.49	0.30	-0.12	0.72
Positive control													
Within-person effects													
On the same day	-0.05	0.95*	0.91	.99	0.35**	0.22	0.48	-0.07	-0.16	0.02	-0.11*	-0.20	-0.01
On the previous day	-0.05	0.96	0.91	1.0	0.22**	0.08	0.36	-0.01	-0.11	0.09	-0.05	-0.18	0.08
Between-person effects	-0.19	0.83	0.60	1.14	0.40*	0.06	0.74	-0.09	-0.32	0.14	0.19	-0.18	0.57
			95% *CI*			95% *CI*			95% *CI*			95% *CI*	
Random Effects (variances)[a]	*Est.*		*LL*	*UL*	*Est.*	*LL*	*UL*	*Est.*	*LL*	*UL*	*Est.*	*LL*	*UL*
Level 2													
Intercept	1.58**		1.06	2.37	0.71	0.18	2.76	-			1.58**	0.82	3.03
Time	.02		.01	.11	0.27	0.06	1.22	0.48**	0.26	0.87	0.67**	0.34	1.30
Previous day outcome	.07*		.03	.16	0.01	0.001	0.43	-			0.08*	0.03	0.20

Negative control												
… On the same day	.01	.002	.04	-			0.03	0.01	0.14	-		
… On the previous day	.01	.00	.06	-			0.02	0.003	0.10	-		
Positive control												
… on the same day	.004	.00	.05	-			-			-		
… on the previous day	.01	.00	.05	-			-			0.06	0.02	0.16
Level 1 [b]												
Residual	1.69**	1.44	1.99	1.05**	0.83	1.32	0.59**	0.49	0.70	1.02**	0.83	1.27
Autocorrelation	.37**	.25	.48	0.30**	0.12	0.46	0.10	-0.10	0.29	0.40**	0.25	0.54

Note. For daily number of cigarettes smoked: *n* = 70 individuals, *n* = 1186 available days; for affect: *n* = 37 individuals; *n* = 353 available days; for doing the opposite: *n* = 38 individuals, *n* = 357 available days; hiding: *n* = 48 individuals, *n* = 530 available days; *RR* = rate ratio; *b* = unstandardized regression coefficients (outcome in original metric), β = standardized regression coefficients (predictor and outcome in between-person *SD* units), 95% *CI* = 95% confidence interval; *LL* = lower level; *UL* = upper level; [a] in some models, some of the random effects could not be computed due to non-convergence. [b] As indicated by the significant level-1 random effects unexplained daily variance, and autocorrelated residuals were present in the sample. * $p < .05$, ** $p < .01$.

Table S1-2

Study 2 Men: Within- and Between-Person Effects of Negative and Positive Social Control on Daily Number of Cigarettes Smoked, Affect, Doing the Opposite, and Hiding Smoking After the Quit Date for Relapsing Smokers Including Random Effects

	DV: Number of cigarettes smoked				DV: Affect			DV: Doing the opposite			DV: Hiding smoking		
			95% *CI*			95% *CI*			95% *CI*			95% *CI*	
Fixed Effects	*b*	*RR*	*LL*	*UL*	β	*LL*	*UL*	*β*	*LL*	*UL*	*β*	*LL*	*UL*
Intercept	1.23	3.43**	2.42	4.86	0.38*	0.03	0.73	1.82**	1.51	2.13	2.03**	1.70	2.36
Time	.07	1.07	.94	1.22	-0.14	-0.38	0.10	0.06	-0.06	0.17	-0.03	-0.22	0.16
Previous day outcome	-.02	.98	.91	1.06	-0.21**	-0.30	-0.13	-0.20**	-0.33	-0.08	-0.28**	-0.37	-0.18
Negative Control													
Within-person effects													
On the same day	.01	1.01	.98	1.05	-0.18*	-0.32	-0.03	0.30**	0.22	0.38	0.24**	0.08	0.39
On the previous day	-.004	1.00	.97	1.02	-0.04	-0.11	0.03	0.04	-0.05	0.12	0.05	-0.03	0.13
Between-person effects	-.11	.90	.72	1.11	-0.05	-0.29	0.18	0.17	-0.11	0.46	0.74**	0.54	0.94
Positive control													
Within-person effects													
On the same day	-.05	.96*	.92	.99	0.22*	0.05	0.39	-0.08	-0.23	0.07	-0.10	-0.27	0.07
On the previous day	-.02	.98	.96	1.0	0.09*	0.01	0.17	-0.06	-0.15	0.04	-0.07	-0.16	0.02
Between-person effects	.22	1.24	.93	1.66	0.30*	0.04	0.56	0.55**	0.25	0.84	-0.16	-0.37	0.04
			95% *CI*			95% *CI*			95% *CI*			95% *CI*	
Random Effects (variances)[a]	*Est.*		*LL*	*UL*	*Est.*	*LL*	*UL*	*Est.*	*LL*	*UL*	*Est.*	*LL*	*UL*
Level 2													
Intercept	1.48**		.90	2.46	0.50	0.15	1.61	0.73**	0.44	1.19	0.72*	0.30	1.71
Time	.14**		.07	.30	0.23	0.08	0.65	-			0.21	0.07	0.58
Previous day outcome	-				-			0.05*	0.02	0.13	0.02	0.004	0.08
Negative control													
… On the same day	-				0.07*	0.03	0.17	-			0.11**	0.05	0.22

… On the previous day	-			-			-			-		
Positive control												
… on the same day	-			0.14*	0.06	0.31	0.08	0.03	0.26	0.17**	0.09	0.35
… on the previous day	-			-			-			-		
Level 1[b]												
Residual	1.41**	1.06	1.87	0.84**	0.60	1.17	0.90**	0.75	1.08	1.21**	0.97	1.52
Autocorrelation	.47**	.28	.63	0.57**	0.39	0.71	0.24*	-0.01	0.45	0.53**	0.40	0.64

Note. For daily number of cigarettes smoked: $n = 60$ men, $n = 945$ available days; for affect: $n = 38$ men; $n = 480$ available days; for doing the opposite $n = 49$ men, $n = 620$ available days; for hiding: $n = 56$ men, $n = 777$ available days; *RR* = rate ratio *b* = unstandardized regression coefficients (outcome in original metric), β = standardized regression coefficients (predictor and outcome in between-person *SD* units), 95% *CI* = 95% confidence interval; *LL* = lower level; *UL* = upper level; [a] in some models, some of the random effects could not be computed due to non-convergence. [b] As indicated by the significant level-1 random effects unexplained daily variance, and autocorrelated residuals were present in the sample; * $p < .05$, ** $p < .01$.

Table S1-3

Study 2 Women: Within- and Between-Person Effects of Negative and Positive Social Control on Daily Number of Cigarettes Smoked, Affect, Doing the Opposite, and Hiding Smoking After the Quit Date for Relapsing Smokers Including Random Effects

	DV: Number of cigarettes smoked				DV: Affect			DV: Doing the opposite			DV: Hiding smoking		
			95% *CI*			95% *CI*			95% *CI*			95% *CI*	
Fixed Effects	*b*	*RR*	*LL*	*UL*	β	*LL*	*UL*	β	*LL*	*UL*	β	*LL*	*UL*
Intercept	1.19	3.28**	2.27	4.74	0.26*	0.03	0.49	1.22**	0.98	1.45	2.89**	2.62	3.15
Time	-.05	.96	.84	1.10	-0.05	-0.15	0.05	0.09	-0.02	0.19	0.02	-0.17	0.22
Previous day outcome	-.03	.97	.91	1.03	-0.02	-0.11	0.08	-0.07*	-0.13	-0.01	-0.15*	-0.27	-0.03
Negative Control													
Within-person effects													
On the same day	.01	1.0	.98	1.04	-0.09	-0.20	0.03	0.09	-0.04	0.22	0.16*	0.001	0.32
On the previous day	.01	1.0	.98	1.03	0.002	-0.05	0.05	0.02	-0.02	0.07	0.06	-0.003	0.13
Between-person effects	-.17	.85	.66	1.09	-0.15	-0.32	0.02	0.02	-0.17	0.22	0.48**	0.25	0.71
Positive control													
Within-person effects													
On the same day	-.03	.98	.94	1.01	0.37**	0.21	0.53	0.01	-0.06	0.07	-0.01	-0.09	0.08
On the previous day	-.03	.97	.92	1.03	0.01	-0.07	0.10	-0.02	-0.09	0.04	0.04	-0.04	0.12
Between-person effects	.23	1.26	.99	1.61	0.28**	0.12	0.45	0.12	-0.08	0.31	-0.14	-0.35	0.07
			95% *CI*			95% *CI*			95% *CI*			95% *CI*	
Random Effects (variances)[a]	*Est.*		*LL*	*UL*	*Est.*	*LL*	*UL*	*Est.*	*LL*	*UL*	*Est.*	*LL*	*UL*
Level 2													
Intercept	1.78**		1.13	2.82	0.19*	0.09	0.41	0.36**	0.22	0.59	0.44*	0.17	1.12
Time	.16*		.07	.40	-			-			0.30*	0.14	0.64
Previous day outcome	.003		.0001	.28	-			-			0.05*	0.02	0.10
Negative control													
… On the same day	-		-		0.02	0.01	0.10	0.07*	0.03	0.16	0.12*	0.05	0.25

… On the previous day	.02	.003	.07	-			-			-		
Positive control												
… on the same day	-	-		0.09*	0.03	0.22	-			-		
… on the previous day	-	-		-			-			-		
Level 1[b]												
Residual	1.47**	1.25	1.74	0.37**	0.31	0.44	0.60**	0.50	0.71	1.11**	0.93	1.33
Autocorrelation	.30**	.16	.42	0.05	-0.19	0.29	0.41**	0.29	0.52	0.27*	0.05	0.46

Note. For daily number of cigarettes smoked: n = 59 women, n = 986 available days; for affect: n = 32 women; n = 336 available days; for doing the opposite: n = 50 women, n = 606 available days; for hiding: n = 55 women, n = 820 available days; *RR* = rate ratio b = unstandardized regression coefficients (outcome in original metric), β = standardized regression coefficients (predictor and outcome in between-person *SD* units), 95% *CI* = 95% confidence interval; *LL* = lower level; *UL* = upper level; [a] in some model, some of the random effects could not be computed due to non-convergence. [b] As indicated by the significant level-1 random effects unexplained daily variance, and autocorrelated residuals were present in the sample; * $p < .05$, ** $p < .01$

Table S1-4

Study 3: Within- and Between-Person Effects of Negative and Positive Social Control on Physical Activity (MVPA), Affect, Doing the Opposite, and Hiding Inactivity Including Random Effects

	DV: MVPA			DV: Affect			DV: Doing the opposite			DV: Hiding inactivity		
		95% *CI*			95% *CI*			95% *CI*			95% *CI*	
Fixed Effects	β	*LL*	*UL*	β	*LL*	*UL*	β	*LL*	*UL*	β	*LL*	*UL*
Intercept	6.10**	5.82	6.37	0.50**	0.25	0.74	2.93**	2.69	3.17	3.23**	3.02	3.44
Time	-0.03	-0.14	0.07	0.005	-0.12	0.13	-0.01	-0.10	0.07	0.04	-0.03	0.11
intervention phase	-0.08	-0.31	0.15	0.10	-0.17	0.36	-0.10	-0.28	0.07	-0.13	-0.27	0.01
experimental group	0.16	-0.21	0.53	-0.22	-0.51	0.06	0.13	-0.16	0.43	-0.04	-0.32	0.23
Within-person weartime	0.09**	0.05	0.13	-			-			-		
Between-person weartime	-0.04	-0.23	0.15	-			-			-		
Previous day outcome	-0.17**	-0.22	-0.12	-0.08*	-0.14	-0.01	-0.04	-0.10	0.01	-0.08*	-0.14	-0.01
Negative Control												
Within-person effects												
On the same day	-0.04	-0.10	0.03	-0.23**	-0.36	-0.10	0.14**	0.05	0.23	0.11**	0.07	0.15
On the previous day	-0.01	-0.07	0.05	-0.04	-0.10	0.03	-0.01	-0.06	0.05	0.06**	0.03	0.10
Between-person effects	-0.01	-0.24	0.22	-0.18*	-0.33	-0.03	0.60**	0.43	0.77	0.49**	0.34	0.65
Positive control												
Within-person effects												
On the same day	0.14**	0.07	0.21	0.57**	0.44	0.69	-0.001	-0.08	0.07	0.005	-0.05	0.06
On the previous day	0.02	-0.03	0.07	-0.001	-0.07	0.07	-0.004	-0.05	0.04	0.004	-0.03	0.04
Between-person effects	-0.09	-0.30	0.12	0.47**	0.31	0.63	0.07	-0.11	0.25	0.02	-0.15	
		95% *CI*			95% *CI*			95% *CI*			95% *CI*	
Random Effects (variances)[a]	*Est.*	*LL*	*UL*	*Est.*	*LL*	*UL*	*Est.*	*LL*	*UL*	*Est.*	*LL*	*UL*
Level 2												
Intercept	0.87**	0.64	1.18	0.46**	0.30	0.70	0.79**	0.56	1.12	0.58**	0.41	0.80
Time	-			-			0.03*	0.02	0.07	0.02*	0.01	0.04

Weartime accelerometer	0.005	0.0002	0.09	-			-			-		
Previous day outcome	0.02**	0.01	0.04	0.04**	0.02	0.07	0.03**	0.01	0.05	0.05**	0.03	0.08
Negative control												
… On the same day	-			0.14**	0.07	0.29	0.09**	0.05	0.16	-		
… On the previous day	-			-			0.01	0.003	0.03	-		
Positive control												
… on the same day	0.04*	0.02	0.08	0.19**	0.11	0.31	0.07**	0.04	0.13	0.03*	0.01	0.06
… on the previous day	-			0.01	0.002	0.09	-			-		
Level 1 [b]												
Residual	1.53**	1.42	1.66	1.52**	1.38	1.66	1.14**	1.07	1.22	0.77**	0.72	0.83
Autocorrelation	0.21**	0.12	0.30	0.16*	0.03	0.29	0.13**	0.04	0.22	0.13*	0.02	0.24

Note. For MVPA: n = 117 individuals, n = 2326 available days; for affect: n = 101 individuals; n = 1697 available days; for doing the opposite n = 120 individuals, n = 2918 available days; hiding: n = 120 individuals, n = 2918 available days; intervention phase: intervention = 0; follow-up phase = 1; experimental group: control group = 0, experimental group = 1; β = standardized regression coefficients (predictor and outcome in between-person *SD* units), 95% *CI* = 95% confidence interval; *LL* = lower level; *UL* = upper level; [a] in some model, some of the random effects could not be computed due to non-convergence. [b] As indicated by the significant level-1 random effects unexplained daily variance, and autocorrelated residuals were present in the sample; * $p < .05$, ** $p < .01$.

Supplemental Material S2

Table S2-1

Study 1: Sensitivity Analyses: Within- and Between-Person Effects of Negative and Positive Social Control on Daily Number of Cigarettes Smoked, Affect, Doing the Opposite, and Hiding Smoking After the Quit Date for Relapsing Smokers Including Random Effects and Control Variables

	DV: Number of cigarettes smoked				DV: Affect			DV: Doing the opposite			DV: Hiding smoking		
			95% *CI*			95% *CI*			95% *CI*			95% *CI*	
Fixed Effects	*b*	*RR*	*LL*	*UL*	β	*LL*	*UL*	β	*LL*	*UL*	β	*LL*	*UL*
Intercept	1.17	3.24**	2.24	4.68	0.06	-0.59	0.72	1.18**	0.84	1.51	2.06**	1.45	2.68
Age	0.01	1.0	0.98	1.03	-0.002	-0.03	0.03	-0.01	-0.03	0.002	-0.02	-0.05	0.01
Gender	0.24	1.27	0.74	2.18	0.18	-0.50	0.86	0.14	-0.24	0.51	-0.34	-0.92	0.25
Nicotine dependence	0.29	1.34**	1.15	1.56	0.01	-0.14	0.17	0.02	-0.06	0.10	0.06	-0.07	0.19
Time	0.10	1.10**	1.03	1.18	-0.05	-0.34	0.24	0.43**	0.14	0.71	0.07	-0.24	0.38
Previous day outcome	0.01	1.01	0.90	1.13	-0.34**	-0.49	-0.20	-0.30**	-0.40	-0.20	-0.29**	-0.45	-0.12
Negative control													
Within-person effects													
On the same day	0.02	1.02	.97	1.08	-0.30**	-0.41	-0.19	0.19*	0.05	0.32	0.11*	0.01	0.20
On the previous day	0.01	1.00	.95	1.05	-0.10	-0.21	0.02	-0.002	-0.12	0.12	0.07	-0.03	0.17
Between-person effects	0.32	1.37**	1.11	1.70	-0.22	-0.67	0.23	0.13	-0.16	0.42	0.29	-0.14	0.72
Positive control													
Within-person effects													
On the same day	-0.05	0.95*	0.91	0.997	0.35**	0.22	0.48	-0.07	-0.16	0.02	-0.11*	-0.20	-0.01
On the previous day	-0.05	0.96	0.91	1.00	0.22**	0.08	0.36	-0.005	-0.10	0.09	-0.05	-0.18	0.08
Between-person effects	-0.27	0.76	0.57	1.02	0.42*	0.05	0.79	-0.05	-0.28	0.18	0.14	-0.23	0.52
			95% *CI*			95% *CI*			95% *CI*			95% *CI*	
Random Effects (variances)[a]	*Est.*		*LL*	*UL*	*Est.*	*LL*	*UL*	*Est.*	*LL*	*UL*	*Est.*	*LL*	*UL*

Level 2												
Intercept	1.28**	0.83	1.96	0.73	0.19	2.77	-			1.54**	0.76	3.14
Time	0.02	0.003	0.12	0.25	0.05	1.27	0.46**	0.25	0.85	0.67**	0.34	1.32
Previous day outcome	0.07*	0.03	0.17	0.01	0.001	0.43	-			0.08*	0.03	0.21
Negative control												
… On the same day	0.01	0.002	0.04	-			0.03	0.01	0.15	-		
… On the previous day	0.01	0.000	0.06	-			0.02	0.003	0.10	-		
Positive control												
… on the same day	0.004	0.000	0.05	-			-			-		
… on the previous day	0.004	0.000	0.06	-			-			0.06	0.02	0.16
Level 1 [b]												
Residual	1.71**	1.45	2.03	1.05**	0.83	1.32	0.59**	0.49	0.70	1.03**	0.83	1.29
Autocorrelation	0.37**	0.25	0.49	0.30**	0.12	0.46	0.10	-0.10	0.29	0.41**	0.25	0.54

Note. Age and nicotine dependence were centered at the average for all relapsers, gender was coded 0 for women, 1 for men. For daily number of cigarettes smoked: n = 70 individuals, n = 1186 available days; for affect: n = 37 individuals; n = 353 available days; for doing the opposite: n = 38 individuals, n = 357 available days; hiding: n = 48 individuals, n = 530 available days; *RR* = rate ratio; *b* = unstandardized regression coefficients (outcome in original metric), β = standardized regression coefficients (predictor and outcome in between-person *SD* units), 95% *CI* = 95% confidence interval; *LL* = lower level; *UL* = upper level; [a] in some models, some of the random effects could not be computed due to non-convergence. [b] As indicated by the significant level-1 random effects unexplained daily variance, and autocorrelated residuals were present in the sample. * $p < .05$, ** $p < .01$.

Table S2-2
Study 2 Men, Sensitivity Analyses: Within- and Between-Person Effects of Negative and Positive Social Control on Daily Number of Cigarettes Smoked, Affect, Doing the Opposite, and Hiding Smoking After the Quit Date for Relapsing Smokers Including Random Effects and Control Variables

	DV: Number of cigarettes smoked				DV: Affect			DV: Doing the opposite			DV: Hiding smoking		
			95% *CI*			95% *CI*			95% *CI*			95% *CI*	
Fixed Effects	*b*	*RR*	*LL*	*UL*	β	*LL*	*UL*	β	*LL*	*UL*	β	*LL*	*UL*
Intercept	1.23	3.41**	2.47	4.69	0.38*	0.03	0.74	1.82**	1.50	2.13	2.05**	1.71	2.38
Age	0.03	1.03**	1.01	1.06	-0.01	-0.02	0.01	-0.003	-0.02	0.02	-0.01	-0.02	0.004
Nicotine dependence	0.13	1.14	0.92	1.41	0.01	-0.13	0.15	0.05	-0.12	0.21	-0.02	-0.13	0.08
Time	0.07	1.07	0.94	1.23	-0.13	-0.37	0.10	0.06	-0.06	0.17	-0.02	-0.22	0.17
Previous day outcome	-0.02	0.98	0.91	1.06	-0.21**	-0.30	-0.13	-0.20**	-0.33	-0.08	-0.28**	-0.38	-0.18
Negative Control													
Within-person effects													
On the same day	0.01	1.01	0.98	1.05	-0.18*	-0.33	-0.04	0.30**	0.22	0.38	0.24**	0.08	0.39
On the previous day	-0.004	0.10	0.97	1.02	-0.04	-0.11	0.03	0.04	-0.05	0.12	0.05	-0.03	0.13
Between-person effects	0.15	1.16	0.89	1.51	-0.09	-0.38	0.20	0.17	-0.15	0.49	0.67**	0.44	0.89
Positive control													
Within-person effects													
On the same day	-0.05	0.96*	0.92	.99	0.22*	0.05	0.38	-0.08	-0.23	0.07	-0.09	-0.26	0.08
On the previous day	-0.02	0.98	0.96	1.0	0.09*	0.01	0.17	-0.06	-0.15	0.04	-0.07	-0.16	0.02
Between-person effects	0.00	1.0	0.75	1.34	0.35*	0.03	0.66	0.56**	0.23	0.89	-0.10	-0.32	0.12
			95% *CI*				95% *CI*			95% *CI*			95% *CI*
Random Effects (variances)[a]	*Est.*		*LL*	*UL*	*Est.*	*LL*	*UL*	*Est.*	*LL*	*UL*	*Est.*	*LL*	*UL*
Level 2													
Intercept	1.26**		0.74	2.14	0.51	0.16	1.64	0.76**	0.46	1.25	0.74*	0.31	1.77
Time	0.15**		0.08	0.31	0.23	0.08	0.65	-			0.21	0.07	0.59

Previous day outcome	-			-			0.05*	0.02	0.13	0.02	0.004	0.08
Negative control												
… On the same day	-			0.07*	0.03	0.17	-			0.11**	0.05	0.22
… On the previous day	-			-			-			-		
Positive control												
… on the same day	-			0.14*	0.06	0.31	0.08	0.03	0.26	0.17**	0.09	0.35
… on the previous day	-			-			-			-		
Level 1 [b]												
Residual	1.40**	1.07	1.82	0.84**	0.60	1.17	0.90**	0.75	1.08	1.22**	0.97	1.53
Autocorrelation	0.46**	0.28	0.61	0.57**	0.39	0.71	0.24*	-0.01	0.45	0.53**	0.40	0.64

Note. Age and nicotine dependence were centered at the average for all male relapsers. For daily number of cigarettes smoked: *n* = 60 individuals, *n* = 945 available days; for affect: *n* = 38 individuals; *n* = 480 available days; for doing the opposite: *n* = 49 individuals, *n* = 620 available days; for hiding: *n* = 56 individuals, *n* = 777 available days; *RR* = rate ratio, *b* = unstandardized regression coefficients (outcome in original metric), β = standardized regression coefficients (predictor and outcome in between-person *SD* units), 95% *CI* = 95% confidence interval; *LL* = lower level; *UL* = upper level; [a] in some models, some of the random effects could not be computed due to non-convergence. [b] As indicated by the significant level-1 random effects unexplained daily variance, and autocorrelated residuals were present in the sample. * $p < .05$, ** $p < .01$.

Table S2-3

Study 2 Women, Sensitivity Analyses: Within- and Between-Person Effects of Negative and Positive Social Control on Daily Number of Cigarettes Smoked, Affect, Doing the Opposite, and Hiding Smoking After the Quit Date for Relapsing Smokers Including Random Effects and Control Variables

	DV: Number of cigarettes smoked				DV: Affect			DV: Doing the opposite			DV: Hiding smoking		
			95% *CI*			95% *CI*			95% *CI*			95% *CI*	
Fixed Effects	*b*	*RR*	*LL*	*UL*	β	*LL*	*UL*	β	*LL*	*UL*	β	*LL*	*UL*
Intercept	1.15	3.15**	2.28	4.34	0.23*	0.02	0.44	1.20**	0.96	1.43	2.88**	2.60	3.17
Age	0.04	1.04**	1.02	1.06	-0.003	-0.02	0.01	-0.0003	-0.01	0.01	0.001	-0.02	0.02
Nicotine dependence	0.30	1.34**	1.12	1.61	-0.003	-0.12	0.11	-0.02	-0.15	0.11	-0.05	-0.20	0.10
Time	-0.05	0.95	0.82	1.10	-0.05	-0.15	0.06	0.08	-0.02	0.18	0.02	-0.18	0.23
Previous day outcome	-0.02	0.98	0.92	1.04	-0.03	-0.12	0.07	-0.11**	-0.16	-0.05	-0.17*	-0.29	-0.04
Negative Control													
Within-person effects													
On the same day	0.01	1.01	0.98	1.04	-0.09	-0.21	0.04	0.11*	0.005	0.22	0.17	-0.001	0.34
On the previous day	0.01	1.01	0.98	1.03	-0.002	-0.05	0.05	0.03	-0.02	0.07	0.07	-0.003	0.13
Between-person effects	-0.03	0.97	0.82	1.16	-0.12	-0.28	0.03	0.09	-0.11	0.29	0.50**	0.25	0.76
Positive control													
Within-person effects													
On the same day	-0.03	1.15	0.93	1.01	0.38**	0.20	0.55	0.02	-0.05	0.08	-0.01	-0.10	0.09
On the previous day	-0.03	0.97	0.91	1.04	0.02	-0.06	0.11	0.003	-0.06	0.07	0.04	-0.05	0.13
Between-person effects	0.14	0.97	0.79	1.69	0.11	-0.10	0.31	-0.03	-0.28	0.22	-0.26	-0.55	0.04
			95% *CI*			95% *CI*			95% *CI*			95% *CI*	
Random Effects (variances)[a]	*Est.*		*LL*	*UL*	*Est.*	*LL*	*UL*	*Est.*	*LL*	*UL*	*Est.*	*LL*	*UL*
Level 2													
Intercept	1.34**		0.82	2.20	0.12	0.04	0.33	0.34**	0.20	0.57	0.51*	0.20	1.30
Time	0.20*		0.09	0.43	-			-			0.29*	0.13	0.66

Previous day outcome	0.002	0.00	0.59	-			-			0.05*	0.02	0.11
Negative control												
… On the same day	-			0.02	0.01	0.11	0.04*	0.01	0.10	0.12*	0.05	0.27
… On the previous day	-			-			-			-		
Positive control												
… on the same day	-			0.10*	0.04	0.24	-			-		
… on the previous day	0.02	0.01	0.08	-			-			-		
Level 1 [b]												
Residual	1.50**	1.29	1.75	0.37**	0.31	0.45	0.52**	0.43	0.61	1.17**	0.97	1.41
Autocorrelation	0.28 **	0.16	0.40	0.06	-0.21	0.33	0.43**	0.31	0.54	0.28**	0.06	0.47

Note. Age and nicotine dependence were centered at the average for all female relapsers. For daily number of cigarettes smoked: n = 57 individuals, n = 950 available days; for affect: n = 30 individuals; n = 320 available days; for doing the opposite: n = 48 individuals, n = 570 available days; for hiding: n = 53 individuals, n = 784 available days;; RR = rate ratio, b = unstandardized regression coefficients (outcome in original metric), β = standardized regression coefficients (predictor and outcome in between-person SD units), 95% CI = 95% confidence interval; LL = lower level; UL = upper level; [a] in some models, some of the random effects could not be computed due to non-convergence. [b] As indicated by the significant level-1 random effects unexplained daily variance, and autocorrelated residuals were present in the sample. * $p < .05$, ** $p < .01$

Table S2-4

Study 3, Sensitivity Analyses: Within- and Between-Person Effects of Negative and Positive Social Control on Physical Activity (MVPA), Affect, Doing the Opposite, and Hiding Inactivity Including Random Effects and Control Variables

	DV: MVPA			DV: Affect			DV: Doing the opposite			DV: Hiding inactivity		
		95% *CI*			95% *CI*			95% *CI*			95% *CI*	
Fixed Effects	β	*LL*	*UL*	β	*LL*	*UL*	β	*LL*	*UL*	β	*LL*	*UL*
Intercept	6.22**	5.88	6.55	0.44**	0.15	0.73	3.09**	2.81	3.38	3.29**	3.02	3.56
Time	-0.06	-0.17	0.04	0.02	-0.11	0.14	-0.01	-0.10	0.08	0.05	-0.02	0.11
Gender	-0.17	-0.53	0.19	0.10	-0.21	0.41	-0.24	-0.55	0.06	-0.14	-0.44	0.16
BMI	-0.04*	-0.07	-0.004	-0.01	-0.04	0.02	0.02	-0.003	0.05	-0.01	-0.03	0.02
Age	-0.02**	-0.03	-0.01	-0.0003	-0.01	0.01	0.001	-0.01	0.01	0.000001	-0.01	0.01
Weekend	-0.29**	-0.45	-0.12	-0.01	-0.16	0.13	-0.07	-0.16	0.03	0.02	-0.05	0.10
intervention phase	-0.03	-0.25	0.20	0.11	-0.16	0.37	-0.12	-0.29	0.06	-0.16*	-0.31	-0.02
experimental group	0.30	-0.06	0.66	-0.21	-0.51	0.09	0.08	-0.23	0.39	-0.01	-0.32	0.29
Within-person weartime	0.07**	0.03	0.11	-			-			-		
Between-person weartime	-0.02	-0.20	0.17	-			-			-		
Previous day outcome	-0.15**	-0.20	-0.10	-0.05	-0.12	0.02	-0.02	-0.08	0.04	-0.09*	-0.16	-0.02
Negative Control												
Within-person effects												
On the same day	-0.04	-0.10	0.03	-0.24**	-0.37	-0.10	0.15**	0.05	0.24	0.11*	0.02	0.21
On the previous day	-0.01	-0.07	0.06	-0.03	-0.09	0.03	0.01	-0.04	0.05	0.04*	0.01	0.08
Between-person effects	-0.07	-0.29	0.16	-0.19*	-0.35	-0.03	0.56**	0.38	0.74	0.57**	0.39	0.74
Positive control												
Within-person effects												
On the same day	0.15**	0.08	0.21	0.59**	0.46	0.71	0.01	-0.07	0.09	-0.02	-0.06	0.01
On the previous day	0.02	-0.03	0.07	-0.01	-0.08	0.06	-0.01	-0.06	0.03	0.003	-0.03	0.04
Between-person effects	-0.06	-0.27	0.14	0.48**	0.32	0.65	0.07	-0.11	0.25	0.01	-0.17	0.19
		95% *CI*			95% *CI*			95% *CI*			95% *CI*	
Random Effects (variances)[a]	*Est.*	*LL*	*UL*	*Est.*	*LL*	*UL*	*Est.*	*LL*	*UL*	*Est.*	*LL*	*UL*

Level 2												
Intercept	0.80**	0.58	1.10	0.50**	0.33	0.76	0.79**	0.56	1.12	0.66**	0.47	0.92
Time	-			-			0.03**	0.02	0.07	0.01	0.005	0.04
Weekend	0.28**	0.15	0.53	-			-			-		
Weartime accelerometer	-			-			-			-		
Previous day outcome	0.02**	0.01	0.04	0.04**	0.02	0.07	0.03**	0.01	0.05	0.05**	0.03	0.09
Negative control												
… On the same day	-			0.16**	0.08	0.32	0.09**	0.05	0.17	0.11**	0.07	0.18
… On the previous day	-			-			-			-		
Positive control												
… on the same day	0.03	0.02	0.08	0.20**	0.12	0.33	0.08**	0.05	0.14	-		
… on the previous day	-			0.01	0.003	0.09	-			-		
Level 1 [b]												
Residual	1.46**	1.35	1.57	1.51**	1.38	1.65	1.17**	1.10	1.25	0.73**	0.67	0.78
Autocorrelation	0.18**	0.08	0.27	0.13*	0.002	0.25	0.12*	0.03	0.20	0.18**	0.07	0.28

Note. BMI and age were centered at the sample average; gender was coded 0 for women, 1 for men; weekend was coded 0 for weedays, 1 for weekend; intervention phase was coded 0 for intervention, 1 for follow-up; experimental group was coded 0 for control group, 1 for experimental group; For MVPA: n = 117 individuals, n = 2326 available days; for affect: n = 100 individuals; n = 1625 available days; for doing the opposite n = 119 individuals, n = 2796 available days; hiding: n = 119 individuals, n = 2796 available days; β = standardized regression coefficients (predictor and outcome in between-person *SD* units), 95% *CI* = 95% confidence interval; *LL* = lower level; *UL* = upper level; [a] in some models, some of the random effects could not be computed due to non-convergence. [b] As indicated by the significant level-1 random effects unexplained daily variance, and autocorrelated residuals were present in the sample. * $p < .05$, ** $p < .01$.

Supplemental Material P1

Table P1

Means, Standard Deviations, Ranges Across all Diary Days, and Intraclass Correlations (ICC) of Provided Positive and Negative Social Control Study 1, Study 2 for Women and Men Separately, and Study 3

		*M*between	*SD*between	*SD*within	Range	*ICC*	*N*
Study 1	Provided positive social control	1.51	0.39	0.45	1 - 2.43	0.40	69
	Provided negative social control	1.13	0.23	0.20	1 – 2.08	0.52	69
Study 2	Provided positive social control	1.51	0.44	0.42	1 – 2.49	0.50	59
Women	Provided negative social control	1.13	0.23	0.22	1 – 2.32	0.49	59
Study 2	Provided positive social control	1.51	0.54	0.43	1 – 3.28	0.59	60
Men	Provided negative social control	1.09	0.19	0.19	1 – 1.94	0.46	60
Study 3	Provided positive social control	1.46	0.40	0.43	1 – 3.10	0.45	115
	Provided negative social control	1.10	0.19	0.20	1 – 2.38	0.45	115

Note. N = Number of cases. The ICC (intraclass correlation) stands for the amount of between-person variance in relation to total variance (Bolger & Laurenceau, 2013).

Table P1-1

Study 1: Within- and Between-Person Effects of Negative and Positive Provided Social Control on Daily Number of Cigarettes Smoked, Affect, Doing the Opposite, and Hiding Smoking After the Quit Date for Relapsing Smokers Including Random Effects

	DV: Number of cigarettes smoked				DV: Affect			DV: Doing the opposite			DV: Hiding smoking		
			95% *CI*			95% *CI*			95% *CI*			95% *CI*	
Fixed Effects	*b*	*RR*	*LL*	*UL*	β	*LL*	*UL*	β	*LL*	*UL*	β	*LL*	*UL*
Intercept	1.27	3.55**	2.59	4.87	0.21	-0.33	0.75	1.40**	1.16	1.64	1.76**	1.37	2.15
Time	0.08	1.08*	1.002	1.17	0.05	-0.30	0.39	0.35*	0.03	0.67	0.18	-0.09	0.44
Previous day outcome	-0.10	0.91#	0.80	1.02	-0.25**	-0.40	-0.10	-0.35**	-0.45	-0.25	-0.21**	-0.37	-0.06
Provided negative control													
Within-person effects													
On the same day	0.07	1.07**	1.05	1.10	-0.26**	-0.41	-0.11	0.12*	0.02	0.22	-0.06	-0.18	0.06
On the previous day	0.03	1.04	0.99	1.08	-0.14#	-0.29	0.01	0.05	-0.05	0.14	0.05	-0.07	0.16
Between-person effects	0.23	1.26	0.92	1.71	-0.12	-0.56	0.31	-0.17	-0.46	0.12	0.39#	-0.006	0.79
Provided positive control													
Within-person effects													
On the same day	-0.11	0.89**	0.85	0.94	0.13*	0.001	0.25	-0.12*	-0.22	-0.02	-0.04	-0.13	0.05
On the previous day	-0.07	0.93**	0.90	0.97	0.12*	0.004	0.24	-0.07	-0.15	0.02	-0.04	-0.15	0.07
Between-person effects	-0.14	0.87	0.58	1.30	0.36#	-0.005	0.73	0.21	-0.07	0.48	0.16	-0.21	0.54
			95% *CI*			95% *CI*			95% *CI*			95% *CI*	
Random Effects (variances)[a]	*Est.*		*LL*	*UL*	*Est.*	*LL*	*UL*	*Est.*	*LL*	*UL*	*Est.*	*LL*	*UL*
Level 2													
Intercept	1.39**		0.90	2.13	1.45*	0.64	3.29	0.04	0.00007	18.85	0.92*	0.43	1.97
Time	0.02		0.003	0.15	0.54#	0.20	1.48	0.51**	0.25	1.05	0.39*	0.19	0.84

Previous day outcome	0.08*	0.03	0.18	0.04	0.009	0.20	-			0.06#	0.02	0.17
Provided negative control												
… On the same day	-			-			-			-		
… On the previous day	0.002	0.00007	0.06	-			-			-		
Provided positive control												
… on the same day	0.006	0.001	0.04	-			0.008	0.0006	0.13	-		
… on the previous day	0.001	0.0000002	3.003	-			-			0.03	0.008	0.11
Level 1 [b]												
Residual	1.84**	1.49	2.27	1.09**	0.88	1.35	0.67**	0.54	0.81	1.07**	0.86	1.33
Autocorrelation	0.48**	0.35	0.60	0.23*	0.04	0.41	0.21*	0.006	0.39	0.29**	0.07	0.48

Note. For daily number of cigarettes smoked: n = 68, n = 1078 available days; for affect: n = 35; n = 330 available days; for doing the opposite: n = 35, n = 351 available days; hiding: n = 45, n = 522 available days; *RR* = rate ratio; b = unstandardized regression coefficients (outcome in original metric), β = standardized regression coefficients (predictor and outcome in between-person *SD* units), 95% *CI* = 95% confidence interval; *LL* = lower level; *UL* = upper level; [a] in some models, some of the random effects could not be computed due to non-convergence. [b] As indicated by the significant level-1 random effects unexplained daily variance, and autocorrelated residuals were present in the sample. * $p < .05$, ** $p < .01$.

Table P1-2

Study 2 Men: Within- and Between-Person Effects of Negative and Positive Provided Social Control on Daily Number of Cigarettes Smoked, Affect, Doing the Opposite, and Hiding Smoking After the Quit Date for Relapsing Smokers Including Random Effects

	DV: Number of cigarettes smoked				DV: Affect			DV: Doing the opposite			DV: Hiding smoking		
			95% *CI*			95% *CI*			95% *CI*			95% *CI*	
Fixed Effects	*b*	*RR*	*LL*	*UL*	β	*LL*	*UL*	*β*	*LL*	*UL*	*β*	*LL*	*UL*
Intercept	1.27	3.56**	2.54	5.00	0.30	-0.07	0.67	1.86**	1.48	2.24	2.06**	1.69	2.44
Time	0.06	1.06	0.93	1.21	-0.04	-0.26	0.17	0.04	-0.11	0.20	-0.07	-0.28	0.14
Previous day outcome	-0.05	0.95	0.88	1.03	-0.38**	-0.49	-0.28	-0.36**	-0.48	-0.24	-0.28**	-0.35	-0.21
Provided negative Control													
Within-person effects													
On the same day	0.06	1.07#	0.99	1.14	-0.19**	-0.29	-0.10	0.14**	0.04	0.24	0.09#	-0.006	0.19
On the previous day	0.03	1.03	0.97	1.09	-0.17**	-0.26	-0.08	0.15**	0.05	0.25	0.09	-0.02	0.19
Between-person effects	-0.11	0.90	0.69	1.16	-0.25	-0.58	0.09	-0.08	-0.50	0.33	0.39*	0.04	0.73
Provided positive control													
Within-person effects													
On the same day	-0.04	0.96*	0.93	1.00	0.09	-0.03	0.20	-0.09	-0.23	0.06	-0.02	-0.14	0.09
On the previous day	-0.005	0.10	0.95	1.04	0.10#	-0.005	0.21	-0.06	-0.18	0.06	-0.03	-0.12	0.06
Between-person effects	0.23	1.26#	0.98	1.62	0.27#	-0.04	0.58	0.19	-0.18	0.57	-0.08	-0.32	0.17
			95% *CI*			95% *CI*			95% *CI*			95% *CI*	
Random Effects (variances)[a]	*Est.*		*LL*	*UL*	*Est.*	*LL*	*UL*	*Est.*	*LL*	*UL*	*Est.*	*LL*	*UL*
Level 2													
Intercept	1.34**		0.77	2.33	1.48	0.001	15.02	0.98**	0.59	1.64	0.98*	0.39	2.41
Time	0.13*		0.05	0.30	0.007	0.00	9.03E10	-			0.22#	0.07	0.73
Previous day outcome	-				-			0.036	0.01	0.12	-		

Provided negative control												
… On the same day	-			-			-			-		
… On the previous day	-			-			-			-		
Provided positive control												
… on the same day	-			-			0.03	0.004	0.23	0.02	0.002	0.17
… on the previous day	-			-			-			-		
Level 1 [b]												
Residual	1.53**	1.07	2.20	1.31**	0.91	1.88	1.20**	0.99	1.46	1.38**	1.06	1.81
Autocorrelation	0.54**	0.32	0.70	0.69**	0.56	0.79	0.45**	0.31	0.57	0.56**	0.41	0.68

Note. For daily number of cigarettes smoked: n = 60, n = 863 available days; for affect: n = 38; n = 431 available days; for doing the opposite: n = 49, n = 562 available days; hiding: n = 55, n = 699 available days; *RR* = rate ratio; *b* = unstandardized regression coefficients (outcome in original metric), β = standardized regression coefficients (predictor and outcome in between-person *SD* units), 95% *CI* = 95% confidence interval; *LL* = lower level; *UL* = upper level; [a] in some models, some of the random effects could not be computed due to non-convergence. [b] As indicated by the significant level-1 random effects unexplained daily variance, and autocorrelated residuals were present in the sample. * $p < .05$, ** $p < .01$.

Table P1-3

Study 2 Women: Within- and Between-Person Effects of Negative and Positive Provided Social Control on Daily Number of Cigarettes Smoked, Affect, Doing the Opposite, and Hiding Smoking After the Quit Date for Relapsing Smokers Including Random Effects

	DV: Number of cigarettes smoked				DV: Affect			DV: Doing the opposite			DV: Hiding smoking		
			95% *CI*			95% *CI*			95% *CI*			95% *CI*	
Fixed Effects	*b*	*RR*	*LL*	*UL*	β	*LL*	*UL*	β	*LL*	*UL*	β	*LL*	*UL*
Intercept	1.11	3.03**	2.06	4.47	0.30*	0.04	0.57	1.32**	1.04	1.59	2.77**	2.46	3.08
Time	-0.03	0.97	0.84	1.14	-0.06	-0.18	0.06	0.06	-0.07	0.19	0.09	-0.13	0.32
Previous day outcome	0.01	1.01	0.94	1.09	0.03	-0.19	0.26	-0.12**	-0.20	-0.04	-0.05	-0.17	0.08
Provided negative Control													
Within-person effects													
On the same day	0.05	1.05**	1.02	1.09	-0.03	-0.13	0.08	0.02	-0.06	0.10	0.10*	0.004	0.20
On the previous day	0.04	1.04#	0.10	1.08	0.02	-0.08	0.11	0.01	-0.07	0.09	-0.0008	-0.10	0.10
Between-person effects	-0.14	0.87	0.66	1.15	-0.08	-0.29	0.14	0.04	-0.20	0.27	0.25#	-0.02	0.53
Provided positive control													
Within-person effects													
On the same day	0.006	1.01	0.98	1.04	0.16#	-0.01	0.33	0.04	-0.04	0.12	-0.07	-0.17	0.03
On the previous day	-0.003	1.00	0.96	1.04	0.05	-0.04	0.13	-0.04	-0.12	0.04	0.11*	0.01	0.20
Between-person effects	0.37	1.45*	1.05	2.00	0.25#	-0.01	0.52	0.03	-0.22	0.28	-0.22	-0.50	0.06
			95% *CI*			95% *CI*			95% *CI*			95% *CI*	
Random Effects (variances)[a]	*Est.*		*LL*	*UL*	*Est.*	*LL*	*UL*	*Est.*	*LL*	*UL*	*Est.*	*LL*	*UL*
Level 2													
Intercept	1.95**		1.23	3.09	0.29**	0.15	0.56	-			0.74*	0.33	1.65
Time	0.23**		0.11	0.50	-			-			0.41**	0.21	0.83
Previous day outcome	0.007		0.00	0.17	0.122#	0.04	0.40	-			0.05*	0.02	0.11

Provided negative control												
… On the same day	-			-			-			-		
… On the previous day	-			-			-			-		
Provided positive control												
… on the same day	-			0.10*	0.04	0.23	-			-		
… on the previous day	0.003	0.00	0.05	-			-			-		
Level 1 [b]												
Residual	1.23**	1.04	1.46	0.48**	0.39	0.59	-			1.13**	0.98	1.31
Autocorrelation	0.27**	0.12	0.41	0.08	-0.24	0.38	-			0.12	-0.13	0.36

Note. For daily number of cigarettes smoked: n = 59, n = 844 available days; for affect: n = 33; n = 308 available days; for doing the opposite: n = 50, n = 524 available days; for hiding: n = 55, n = 707 available days; *RR* = rate ratio; b = unstandardized regression coefficients (outcome in original metric), β = standardized regression coefficients (predictor and outcome in between-person *SD* units), 95% *CI* = 95% confidence interval; *LL* = lower level; *UL* = upper level; [a] in some models, some of the random effects could not be computed due to non-convergence. [b] As indicated by the significant level-1 random effects unexplained daily variance, and autocorrelated residuals were present in the sample. * $p < .05$, ** $p < .01$.

Table P1-4

Study 3: Within- and Between-Person Effects of Negative and Positive Provided Social Control on Physical Activity (MVPA), Affect, Doing the Opposite, and Hiding Inactivity Including Random Effects

	DV: MVPA			DV: Affect			DV: Doing the opposite			DV: Hiding inactivity		
		95% *CI*			95% *CI*			95% *CI*			95% *CI*	
Fixed Effects	β	*LL*	*UL*	β	*LL*	*UL*	β	*LL*	*UL*	β	*LL*	*UL*
Intercept	6.09**	5.82	6.37	0.51**	0.22	0.80	2.95**	2.66	3.24	3.20**	2.93	3.47
Time	-0.04	-0.14	0.07	0.04	-0.11	0.18	-0.04	-0.13	0.06	0.03	-0.04	0.10
intervention phase	-0.09	-0.32	0.15	0.09	-0.25	0.42	-0.08	-0.27	0.12	-0.12	-0.27	0.04
experimental group	0.20	-0.16	0.57	-0.20	-0.55	0.15	0.14	-0.24	0.52	0.03	-0.35	0.41
Within-person weartime	0.09**	0.05	0.12	-			-			-		
Between-person weartime	-0.05	-0.23	0.13	-			-			-		
Previous day outcome	-0.19**	-0.24	-0.14	-0.17**	-0.24	-0.10	-0.05#	-0.11	0.007	-0.12**	-0.19	-0.05
Provided negative Control												
Within-person effects												
On the same day	0.006	-0.05	0.06	-0.15**	-0.22	-0.07	0.02	-0.02	0.06	-0.01	-0.05	0.02
On the previous day	-0.02	-0.08	0.03	0.005	-0.07	0.08	0.01	-0.03	0.05	-0.009	-0.04	0.02
Between-person effects	0.09	-0.13	0.31	-0.48**	-0.72	-0.24	-0.004	-0.24	0.23	0.08	-0.16	0.32
Provided positive control												
Within-person effects												
On the same day	0.14**	0.08	0.20	0.39**	0.27	0.50	-0.04#	-0.08	0.003	-0.01	-0.05	0.02
On the previous day	-0.03	-0.08	0.03	0.07*	0.003	0.14	-0.02	-0.07	0.03	-0.02	-0.05	0.02
Between-person effects	-0.13	-0.36	0.10	0.34**	0.12	0.56	0.07	-0.17	0.32	-0.05	-0.29	0.20
		95% *CI*			95% *CI*			95% *CI*			95% *CI*	
Random Effects (variances)[a]	*Est.*	*LL*	*UL*	*Est.*	*LL*	*UL*	*Est.*	*LL*	*UL*	*Est.*	*LL*	*UL*
Level 2												
Intercept	0.84**	0.62	1.14	0.56**	0.34	0.92	1.19**	0.85	1.65	0.92**	0.66	1.26
Time	-			-			0.03*	0.01	0.07	0.01	0.003	0.04

Weartime accelerometer	-			-			-			-		
Previous day outcome	0.02*	0.009	0.05	0.03**	0.02	0.07	0.03**	0.01	0.05	0.05**	0.03	0.08
Provided negative control												
… On the same day	-			-			-			-		
… On the previous day	-			-			-			-		
Provided positive control												
… on the same day	0.01	0.002	0.06	0.14**	0.78	0.25	-			-		
… on the previous day	0.008	0.001	0.08	-			0.01	0.03	0.05	-		
Level 1 [b]												
Residual	1.59**	1.47	1.71	2.00**	1.80	2.22	1.33**	1.24	1.42	0.85**	0.78	0.92
Autocorrelation	0.24**	0.15	0.32	0.26**	0.16	0.36	0.18**	0.09	0.27	0.20**	0.09	0.31

Note. For MVPA: n = 117, n = 2337 available days; for affect: n = 101; n = 1476 available days; for doing the opposite n = 120, n = 2515 available days; hiding: n = 120, n = 2515 available days; intervention phase: intervention = 0; follow-up phase = 1; experimental group: control group = 0, experimental group = 1; β = standardized regression coefficients (predictor and outcome in between-person *SD* units), 95% *CI* = 95% confidence interval; *LL* = lower level; *UL* = upper level; [a] in some models, some of the random effects could not be computed due to non-convergence. [b] As indicated by the significant level-1 random effects unexplained daily variance, and autocorrelated residuals were present in the sample. * $p < .05$, ** $p < .01$.

Supplemental Material P2

Table P2-1

Study 1, Sensitivity Analyses: Within- and Between-Person Effects of Negative and Positive Provided Social Control on Daily Number of Cigarettes Smoked, Affect, Doing the Opposite, and Hiding Smoking After the Quit Date for Relapsing Smokers Including Random Effects and Control Variables

	DV: Number of cigarettes smoked				DV: Affect			DV: Doing the opposite			DV: Hiding smoking		
			95% *CI*			95% *CI*			95% *CI*			95% *CI*	
Fixed Effects	*b*	*RR*	*LL*	*UL*	β	*LL*	*UL*	β	*LL*	*UL*	β	*LL*	*UL*
Intercept	1.20	3.33**	2.33	4.75	0.30	-0.35	0.94	1.35**	1.00	1.70	1.79**	1.22	2.36
Age	-0.02	1.00	0.97	1.03	0.02	-0.01	0.05	-0.03*	-0.04	-0.01	-0.02	-0.05	0.01
Gender	0.23	1.26	0.76	2.09	-0.10	-0.67	0.47	0.02	-0.37	0.40	-0.06	-0.69	0.57
Nicotine dependence	0.30	1.34**	1.16	1.55	0.001	-0.14	0.14	0.07#	-0.01	0.15	0.10	-0.05	0.24
Time	0.08	1.08*	1.00	1.16	0.06	-0.28	0.41	0.31#	-0.2	0.63	0.17	-0.10	0.44
Previous day outcome	-0.10	0.91	0.80	1.02	-0.25**	-0.41	-0.10	-0.37*	-0.46	-0.27	-0.22*	-0.37	-0.06
Provided negative control													
Within-person effects													
On the same day	0.07	1.07**	1.04	1.10	-0.26**	-0.41	-0.12	0.13*	0.03	0.23	-0.06	-0.18	0.06
On the previous day	0.04	1.04#	1.00	1.08	-0.14#	-0.29	0.06	0.06	-0.04	0.16	0.05	-0.07	0.17
Between-person effects	0.15	1.16	0.89	1.51	-0.03	-0.53	0.47	-0.38*	-0.69	-0.08	0.31	-0.10	0.72
Provided positive control													
Within-person effects													
On the same day	-0.11	0.90**	0.85	0.94	0.13*	0.01	0.25	-0.11*	-0.21	-0.01	-0.04	-0.13	0.05
On the previous day	-0.07	0.93**	0.90	0.97	0.12*	0.002	0.24	-0.07#	-0.16	0.01	-0.04	-0.15	0.07
Between-person effects	-0.24	0.79	0.55	1.13	0.39#	-0.01	0.79	0.29*	0.02	0.55	0.14	-0.25	0.52
			95% *CI*			95% *CI*			95% *CI*			95% *CI*	
Random Effects (variances)[a]	*Est.*		*LL*	*UL*	*Est.*	*LL*	*UL*	*Est.*	*LL*	*UL*	*Est.*	*LL*	*UL*

Level 2												
Intercept	1.11**	0.70	1.76	1.46*	0.63	3.37	0.06	0.001	6.12	0.87*	0.38	2.03
Time	0.01	0.001	0.20	0.52#	0.19	1.48	0.54*	0.25	1.14	0.40*	0.19	0.87
Previous day outcome	0.08*	0.03	0.19	0.04	0.01	0.20	-			0.06#	0.02	0.18
Provided negative control												
… On the same day	-			-			-			-		
… On the previous day	-			-			-			-		
Provided positive control												
… on the same day	0.01	0.001	0.04	-			0.01	0.001	0.15	-		
… on the previous day	0.001	0.0001	1.52	-			-			0.03	0.01	0.12
Level 1 [b]												
Residual	1.87**	1.50	2.33	1.09**	0.88	1.35	0.67**	0.55	0.82	1.08**	0.87	1.35
Autocorrelation	0.48**	0.35	0.60	0.23*	0.04	0.41	0.22*	0.02	0.40	0.30*	0.09	0.49

Note. Age and nicotine dependence were centered at the average for all relapsers, gender was coded 0 for women, 1 for men. For daily number of cigarettes smoked: *n* = 68 individuals, *n* = 1078 available days; for affect: *n* = 35 individuals; *n* = 330 available days; for doing the opposite: *n* = 35 individuals, *n* = 351 available days; hiding: *n* = 45 individuals, *n* = 522 available days; *RR* = rate ratio; *b* = unstandardized regression coefficients (outcome in original metric), β = standardized regression coefficients (predictor and outcome in between-person *SD* units), 95% *CI* = 95% confidence interval; *LL* = lower level; *UL* = upper level; [a] in some models, some of the random effects could not be computed due to non-convergence. [b] As indicated by the significant level-1 random effects unexplained daily variance, and autocorrelated residuals were present in the sample. # $p < .10$; * $p < .05$, ** $p < .01$.

Table P2-2

Study 2 Men, Sensitivity Analyses: Within- and Between-Person Effects of Negative and Positive Provided Social Control on Daily Number of Cigarettes Smoked, Affect, Doing the Opposite, and Hiding Smoking After the Quit Date for Relapsing Smokers Including Random Effects and Control Variables

	DV: Number of cigarettes smoked				DV: Affect			DV: Doing the opposite			DV: Hiding smoking		
			95% *CI*			95% *CI*			95% *CI*			95% *CI*	
Fixed Effects	*b*	*RR*	*LL*	*UL*	β	*LL*	*UL*	β	*LL*	*UL*	β	*LL*	*UL*
Intercept	1.26	3.54**	2.53	4.95	0.31#	-0.04	0.66	1.85**	1.47	2.24	2.08**	1.70	2.45
Age	0.01	1.01	0.99	1.03	-0.02**	-0.04	-0.006	0.005	-0.02	0.03	0.0003	-0.02	0.02
Nicotine dependence	0.08	1.09	0.91	1.30	-0.14*	-0.25	-0.03	0.03	-0.15	0.20	0.02	-0.13	0.17
Time	0.06	1.06	0.93	1.21	-0.03	-0.24	0.17	0.04	-0.11	0.20	-0.08	-0.28	0.13
Previous day outcome	-0.05	0.95	0.88	1.03	-0.39**	-0.50	-0.28	-0.36**	-0.48	-0.24	-0.29**	-0.36	-0.21
Provided negative Control													
Within-person effects													
On the same day	0.06	1.07#	0.99	1.14	-0.20**	-0.29	-0.11	0.14**	0.04	0.24	0.07	-0.03	0.16
On the previous day	0.03	1.03	0.97	1.09	-0.17**	-0.26	-0.08	0.15**	0.05	0.25	0.09#	-0.01	0.19
Between-person effects	-0.13	0.88	0.69	1.13	-0.20	-0.50	0.10	-0.09	-0.52	0.33	0.40*	0.04	0.76
Provided positive control													
Within-person effects													
On the same day	-0.04	0.96*	0.93	1.00	0.09	-0.03	0.20	-0.09	-0.23	0.06	-0.03	-0.13	0.06
On the previous day	-0.005	1.00	0.95	1.04	0.10#	-0.005	0.21	-0.06	-0.18	0.06	-0.03	-0.12	0.06
Between-person effects	0.20	1.22	0.94	1.59	0.35*	0.07	0.64	0.18	-0.21	0.58	-0.09	-0.35	0.18
			95% *CI*			95% *CI*			95% *CI*			95% *CI*	
Random Effects (variances)[a]	*Est.*		*LL*	*UL*	*Est.*	*LL*	*UL*	*Est.*	*LL*	*UL*	*Est.*	*LL*	*UL*
Level 2													
Intercept	1.38**		0.80	2.37	0.06	0.0009	4.34	1.01**	0.60	1.71	0.99*	0.41	2.42

Time	0.14*	0.06	0.30	-			-			0.20	0.05	0.70
Previous day outcome	-			-			0.04	0.01	0.12	-		
Provided negative control												
… On the same day	-			-			-			-		
… On the previous day	-			-			-			-		
Provided positive control												
… on the same day	-			-			0.03	0.004	0.23	-		
… on the previous day	-			-			-			-		
Level 1 [b]												
Residual	1.52**	1.09	2.13	1.31**	0.98	1.75	1.20**	0.99	1.46	1.39**	1.08	1.80
Autocorrelation	0.53**	0.33	0.69	0.70**	0.59	0.78	0.45**	0.31	0.57	0.55**	0.40	0.67

Note. Age and nicotine dependence were centered at the average for all male relapsers. For daily number of cigarettes smoked: *n* = 60 individuals, *n* = 863 available days; for affect: *n* = 38 individuals; *n* = 431 available days; for doing the opposite: *n* = 49 individuals, *n* = 562 available days; for hiding: *n* = 55 individuals, *n* = 699 available days; *RR* = rate ratio, *b* = unstandardized regression coefficients (outcome in original metric), β = standardized regression coefficients (predictor and outcome in between-person *SD* units), 95% *CI* = 95% confidence interval; *LL* = lower level; *UL* = upper level; [a] in some models, some of the random effects could not be computed due to non-convergence. [b] As indicated by the significant level-1 random effects unexplained daily variance, and autocorrelated residuals were present in the sample. # $p < .10$; * $p < .05$, ** $p < .01$.

Table P2-3

Study 2 Women, Sensitivity Analyses: Within- and Between-Person Effects of Negative and Positive Provided Social Control on Daily Number of Cigarettes Smoked, Affect, Doing the Opposite, and Hiding Smoking After the Quit Date for Relapsing Smokers Including Random Effects and Control Variables

	DV: Number of cigarettes smoked				DV: Affect			DV: Doing the opposite			DV: Hiding smoking		
			95% *CI*			95% *CI*			95% *CI*			95% *CI*	
Fixed Effects	*b*	*RR*	*LL*	*UL*	β	*LL*	*UL*	β	*LL*	*UL*	β	*LL*	*UL*
Intercept	1.06	2.89**	1.94	4.30	0.31*	0.05	0.56	1.32**	1.04	1.59	2.77**	2.45	3.09
Age	0.02	1.02	1.00	1.05	-0.02*	-0.03	-0.001	0.004	-0.01	0.02	-0.0003	-0.02	0.02
Nicotine dependence	0.02	1.02	0.75	1.38	-0.04	-0.19	0.12	0.06	-0.09	0.21	0.04	-0.04	0.21
Time	-0.03	0.98	0.83	1.15	-0.05	-0.17	0.07	0.07	-0.06	0.20	0.10	-0.13	0.32
Previous day outcome	0.02	1.02	0.95	1.11	0.03	-0.19	0.26	-0.12**	-0.20	-0.04	-0.05	-0.18	0.07
Provided negative Control													
Within-person effects													
On the same day	0.05	1.05**	1.02	1.09	-0.03	-0.13	0.08	0.02	-0.05	0.10	0.10*	0.004	0.20
On the previous day	0.04	1.04*	1.002	1.08	0.02	-0.08	0.11	0.01	-0.06	0.09	-0.002	-0.10	0.10
Between-person effects	-0.14	0.87	0.67	1.13	-0.06	-0.26	0.15	0.04	-0.20	0.28	0.26#	-0.02	0.55
Provided positive control													
Within-person effects													
On the same day	0.006	1.006	0.98	1.04	0.17*	0.001	0.34	0.04	-0.04	0.12	-0.07	-0.17	0.04
On the previous day	-0.005	1.00	0.95	1.04	0.05	-0.04	0.13	-0.04	-0.12	0.04	0.11*	0.009	0.20
Between-person effects	0.35	1.42#	1.00	2.03	0.30*	0.04	0.55	0.009	-0.25	0.27	-0.23	-0.53	0.07
			95% *CI*			95% *CI*			95% *CI*			95% *CI*	
Random Effects (variances)[a]	*Est.*		*LL*	*UL*	*Est.*	*LL*	*UL*	*Est.*	*LL*	*UL*	*Est.*	*LL*	*UL*
Level 2													
Intercept	2.03**		1.28	3.24	0.25**	0.12	0.51	-			0.80*	0.37	1.80

Time	0.26**	0.13	0.54	-			-			0.42**	0.21	0.84
Previous day outcome	0.009	0.00	0.19	0.12#	0.04	0.40	-			0.05*	0.02	0.11
Provided negative control												
… On the same day	-			-			-			-		
… On the previous day	-			-			-			-		
Provided positive control												
… on the same day	-			0.10*	0.04	0.24	-			-		
… on the previous day	0.003	0.00	0.05	-			-			-		
Level 1 [b]												
Residual	1.20**	1.03	1.41	0.48**	0.39	0.59	-			1.14**	0.98	1.31
Autocorrelation	0.25**	0.11	0.38	0.08	-0.23	0.38	-			0.13	-0.12	0.37

Note. Age and nicotine dependence were centered at the average for all female relapsers. For daily number of cigarettes smoked: *n* = 58 individuals, *n* = 824 available days; for affect: *n* = 33 individuals; *n* = 308 available days; for doing the opposite: *n* = 49 individuals, *n* = 523 available days; for hiding: *n* = 54 individuals, *n* = 706 available days; *RR* = rate ratio, *b* = unstandardized regression coefficients (outcome in original metric), β = standardized regression coefficients (predictor and outcome in between-person *SD* units), 95% *CI* = 95% confidence interval; *LL* = lower level; *UL* = upper level; [a] in some models, some of the random effects could not be computed due to non-convergence. [b] As indicated by the significant level-1 random effects unexplained daily variance, and autocorrelated residuals were present in the sample. # $p < .10$; * $p < .05$, ** $p < .01$.

Table P2-4

Study 3, Sensitivity Analyses: Within- and Between-Person Effects of Negative and Positive Provided Social Control on Physical Activity (MVPA), Affect, Doing the Opposite, and Hiding Inactivity Including Random Effects and Control Variables

	DV: MVPA			DV: Affect			DV: Doing the opposite			DV: Hiding inactivity		
		95% *CI*			95% *CI*			95% *CI*			95% *CI*	
Fixed Effects	β	*LL*	*UL*	β	*LL*	*UL*	β	*LL*	*UL*	β	*LL*	*UL*
Intercept	6.23**	5.91	6.56	0.42*	0.07	0.76	2.99**	2.64	3.34	3.17**	2.84	3.50
Time	-0.06	-0.17	0.04	0.05	-0.10	0.20	-0.03	-0.12	0.07	0.04	-0.04	0.12
Gender	-0.22	-0.57	0.13	0.14	-0.22	0.50	-0.07	-0.45	0.31	0.05	-0.33	0.42
BMI	-0.03*	-0.07	-0.001	-0.01	-0.04	0.02	0.01	-0.02	0.05	-0.02	0.05	0.02
Age	-0.02**	-0.03	-0.008	-0.007	-0.02	0.007	-0.005	-0.02	0.009	-0.003	-0.02	0.01
Weekend	-0.26**	-0.42	-0.09	0.07	-0.09	0.24	-0.04	-0.14	0.07	-0.010	-0.09	0.07
intervention phase	-0.03	-0.26	0.20	0.09	-0.25	0.43	-0.09	-0.29	0.10	-0.13	-0.29	0.03
experimental group	0.33#	-0.03	0.69	-0.16	-0.52	0.21	0.14	-0.25	0.53	0.05	-0.34	0.44
Within-person weartime	0.06**	0.02	0.10	-			-			-		
Between-person weartime	-0.02	-0.20	0.16	-			-			-		
Previous day outcome	-0.18**	-0.23	-0.13	-0.15**	-0.22	-0.08	-0.04	-0.10	0.02	-0.13**	-0.20	-0.06
Provided negative Control												
Within-person effects												
On the same day	0.0005	-0.05	0.06	-0.15**	-0.23	-0.08	0.02	-0.02	0.06	-0.01	-0.05	0.03
On the previous day	-0.003	-0.06	0.05	0.001	-0.07	0.08	0.008	-0.04	0.05	-0.009	-0.04	0.03
Between-person effects	0.05	-0.18	0.27	-0.50**	-0.76	-0.25	-0.02	-0.26	0.23	0.06	-0.19	0.31
Provided positive control												
Within-person effects												
On the same day	0.15**	0.10	0.21	0.40**	0.28	0.51	-0.04#	-0.09	0.004	-0.01	-0.05	0.02
On the previous day	-0.03	-0.08	0.02	0.05	-0.02	0.13	-0.02	-0.07	0.03	-0.02	-0.05	0.02
Between-person effects	-0.07	-0.30	0.16	0.35**	0.12	0.58	0.08	-0.17	0.34	-0.02	-0.27	0.24
		95% *CI*			95% *CI*			95% *CI*			95% *CI*	
Random Effects (variances)[a]	*Est.*	*LL*	*UL*	*Est.*	*LL*	*UL*	*Est.*	*LL*	*UL*	*Est.*	*LL*	*UL*

Level 2												
Intercept	0.75**	0.54	1.05	0.60**	0.36	0.98	1.22**	0.87	1.71	0.91**	0.65	1.27
Time	-			-			0.03*	0.01	0.08	0.01	0.003	0.05
Weekend	0.29**	0.15	0.55	-			-			-		
Weartime accelerometer	-			-			-			-		
Previous day outcome	0.02*	0.01	0.05	0.04**	0.02	0.07	0.03**	0.02	0.06	0.05**	0.03	0.09
Provided negative control												
… On the same day	-			-			-			-		
… On the previous day	-			-			-			-		
Provided positive control												
… on the same day	0.006	0.0004	0.10	0.15**	0.08	0.26	-			-		
… on the previous day	-			-			0.01	0.004	0.05	-		
Level 1 [b]												
Residual	1.52**	1.40	1.64	2.00**	1.81	2.22	1.36**	1.27	1.46	0.86**	0.79	0.94
Autocorrelation	0.22**	0.13	0.31	0.25**	0.15	0.35	0.17**	0.07	0.26	0.22**	0.11	0.32

Note. BMI and age were centered at the sample average; gender was coded 0 for women, 1 for men; weekend was coded 0 for weedays, 1 for weekend; intervention phase was coded 0 for intervention, 1 for follow-up; experimental group was coded 0 for control group, 1 for experimental group; For MVPA: n = 117 individuals, n = 2337 available days; for affect: n = 100 individuals; n = 1476 available days; for doing the opposite n = 119 individuals, n = 2515 available days; hiding: n = 119 individuals, n = 2515 available days; β = standardized regression coefficients (predictor and outcome in between-person *SD* units), 95% *CI* = 95% confidence interval; *LL* = lower level; *UL* = upper level; [a] in some models, some of the random effects could not be computed due to non-convergence. [b] As indicated by the significant level-1 random effects unexplained daily variance, and autocorrelated residuals were present in the sample. # $p < .10$; * $p < .05$, ** $p < .01$.

Supplemental Material Table P6

Table P6

Overview of Results for Study 1, Study 2 (for Men and Women), and Study 3 for partner-reported provided positive and negative social control

	Target behavior				Affect				Reactance-related responses							
	Smoking			Activity					Doing the opposite				Hiding			
	Study1	Study2		Study3	Study1	Study2		Study3	Study1	Study2		Study3	Study1	Study2		Study3
		Men	Women			Men	Women			Men	Women			Men	Women	
**Provided negative control**																
Within-person same day	+	+#	+	0	- -	-	0	-	+#	+	0	0	0	+#	+	0
Within person previous day	0	0	+#	0	-#	-	0	0	0	+	0	0	0	0	0	0
**Provided positive control**																
Within-person same day	-	-	0	+	+ +	0	+ #	+ +	-	00	0	-#	0	0	0	0
Within person previous day	-	0	0	0	+	+#	0	+	0	0	0	0	0	0	+	0

Note. 0 = non-significant effect; + = positive significant effect; - = negative significant effect; + or - = small effect size, ++ or -- = medium effect size, +++ or --- = large effect size; effect sizes are only displayed for significant effects; red colour coding: disconfirming hypothesis, green colour coding: confirming hypothesis; white colour coding: no a priori hypotheses.
